# Discovering the Anti-Inflammatory Potential of Compounds Isolated from the Aerial Parts of *Gelasia tomentosa* (L.) Zaika, Sukhor. & N.Kilian (Syn. *Scorzonera tomentosa*), Through In Vitro Techniques and Advanced In Silico Modeling Approaches

**DOI:** 10.3390/molecules31010019

**Published:** 2025-12-20

**Authors:** Özlem Bahadır-Acıkara, Beyzanur Akcan, Sezen Yılmaz-Sarıaltın, Christian Zidorn

**Affiliations:** 1 Department of Pharmacognosy, Faculty of Pharmacy, Ankara University, Tandogan, Ankara 06560, Turkey; akcanbeyzanur123@gmail.com; 2 Department of Pharmaceutical Toxicology, Faculty of Pharmacy, Ankara University, Tandogan, Ankara 06560, Turkey; sezen.yilmaz@ankara.edu.tr; 3 Abteilung Pharmazeutische Biologie, Pharmazeutisches Institut, Christian-Albrechts-Universität zu Kiel, Gutenbergstraße 76, 24118 Kiel, Germany; czidorn@pharmazie.uni-kiel.de; 4 Division of Pharmaceutical Biotechnology, Department of Pharmaceutical Biology and Biotechnology, Wroclaw Medical University, Borowska 211, 50-556 Wrocław, Poland

**Keywords:** anti-inflammatory activity, caffeoylquinic acid, flavonoid, *Gelasia tomentosa*, in silico, *Scorzonera*, *Scorzonera tomentosa*

## Abstract

*Gelasia tomentosa* (L.) Zaika, Sukhor. & N.Kilian which is known formerly as *Scorzonera tomentosa* L., a wild edible plant species in Turkey, is traditionally used against rheumatism and for wound healing. In this study, we explore its anti-inflammatory compounds, evaluating effectiveness through human red blood cell stabilization and in silico models, alongside physico-chemical and pharmacokinetic profiles. In vitro activity-guided fractionation allowed the isolation of sixteen compounds from the aerial parts of *G. tomentosa*, which were identified as hyperoside (**1**), isoquercetin (**2**), quercetin 3-*O*-*β*-apiofuranosyl-(1→2)-*β*-galactopyranoside (**3**), quercetin 3-*O*-*β*-apiofuranosyl-(1→2)-*β*-glucopyranoside (**4**), 7-methoxyapigenin-6-*C*-*β*-apiofuranosyl-(1→2)-*β*-glucopyranoside (**5**), apigenin-6-*C*-*β*-apiofuranosyl-(1→2)-*β*-glucopyranoside (**6**), dihydrodehydrodiconiferyl-alcohol-4-*O*-*β*-glucopyranoside (**7**), cichoriin (**8**), 7-*O*-methylisoorientin (**9**), isoorientin (**10**), swertisin (**11**), 3,5-*O*-dicaffeoylquinic acid methyl ester (**12**), 4,5-*O*-dicaffeoylquinic acid methyl ester (**13**), staphylinioside E (**14**), 3,5-*O*-dicaffeoylquinic acid (**15**), and 4,5-*O*-dicaffeoylquinic acid (**16**). Compound 16 displayed the highest potential anti-inflammatory activity (IC_50_ = 0.55 ± 0.00 mg/mL). However, the fraction with compounds displayed stronger biological activity than the isolated ones. In silico findings supported the anti-inflammatory potential, enhancing TP53 expression and cell membrane protection. Cichoriin (**8**) and staphylinioside E (**14**) are in accordance with Lipinski’s, Pfizer’s, GSK’s, and Golden Triangle rules, indicating a favorable ADME profile as a drug candidate. Further studies are needed to test this potential in specific inflammation models.

## 1. Introduction

Inflammation, characterized by a variety of physiological and immunological processes regulated mainly by cytokines, can be described as a multifaceted nonspecific immune reaction of the body to harmful stimuli, pathogens, and irritating chemicals [1,2,3,4]. Acute inflammation is a necessary first response to a harmful stimulus. A normal inflammatory response can be controlled by suppressing proinflammatory proteins and increasing the expression of anti-inflammatory proteins, which ultimately leads to the termination of the inflammation process and the return of damaged tissue to its normal state, thereby regaining its former functions [4]. However, when inflammation cannot be controlled or persists for too long, it becomes chronic, causing tissue damage and potentially leading to certain chronic metabolic diseases [5]. Experimental evidence has demonstrated that chronic inflammation plays a significant role in the development of various chronic diseases, including cancer, diabetes, obesity, and arthritis, as well as cardiovascular, neurological, pulmonary, psychological, and autoimmune disorders. These conditions can severely diminish the quality of life and even lead to mortality [6]. Numerous scientific studies have established a link between chronic inflammation and cancer, characterized by the infiltration of mononuclear immune cells, tissue destruction, fibrosis, increased angiogenesis, and enhanced DNA synthesis and cell proliferation. This process disrupts DNA repair mechanisms and inhibits apoptosis, which can contribute to the onset and progression of cancer. Additionally, elevated levels of cytokines, proinflammatory molecules, inducible nitric oxide synthase, reactive oxygen species (ROS), and Nuclear Factor kappa (NF-κB) associated with chronic inflammation foster a microenvironment conducive to malignant cell growth, promoting mutations and tumor development [7]. The wound-healing process consists of four different stages, one of which is inflammation. The inflammation phase starts immediately following injury, together with hemostasis, and is characterized by a variety of cascades, including blood vessel constriction, platelet activation, and aggregation, which result in the secretion of a large number of cytokines and the promotion of neutrophils and macrophages, allowing the progression of the inflammatory phase [8]. This series of events leads to wound closure and the restoration of normal skin integrity. Wound-healing drugs have challenging tasks in terms of maintaining balance during the inflammatory process. Secondary metabolites can modulate inflammation by acting on cells, growth factors, and cytokines involved in wound healing, which results in promoted angiogenesis, fibroplasia, and epithelization [9]. Consequently, addressing inflammation and exploring compounds with anti-inflammatory properties is of considerable importance [7].

*Gelasia tomentosa* (L.) Zaika, Sukhor. & N.Kilian, which is formerly known as *Scorzonera tomentosa* L., is a perennial species with cylindrical roots and simple leaves in lanceolate or ovate form, grows naturally in Turkey [10], and the stems of the plant are recorded as wild edible plants after peeling [11]. The genus *Scorzonera* is recorded in Turkish folk medicine as medicinal plants which are used for treating rheumatism, arteriosclerosis, kidney diseases, hypertension, and diabetes mellitus [12]. The plant roots and their latex are used for their wound-healing properties mainly in the middle and eastern parts of Anatolia [13], as well as in the treatment of women’s infertility and stomach ailments in Turkish folk medicine [14]. It has also been recorded that the roots of the plant are beneficial because of their hemostatic activity [15], and the leaves are reported for their treating activities against fungal infections in the east region and southeast part of Anatolia, respectively [16].

Anti-inflammatory [17], analgesic [18], and hepatoprotective [19] activities of *G. tomentosa* in vivo and potential inhibitory effects on NF-κB nuclear translocation, tumor necrosis factor (TNF)-α, and interleukin (IL)-1*β* production in vitro were reported in previous research [20]. Further studies regarding the inhibitory activities of *G. tomentosa* on acetylcholinesterase and butyrylcholinesterase as well as against tyrosinase α-amylase enzymes, together with its antioxidant capacity, have been published recently [21,22]. *G. tomentosa* displayed significant wound healing activity in the linear incision and circular excision wound models in vivo, and, in particular, the aerial parts of the plant activity were found to be remarkable as follows: 25.4% and 37.62% in both the linear incision and circular excision wound models, respectively [17]. It has been suggested that the wound-healing activity of *G. tomentosa* is based on its anti-inflammatory activity, which has been confirmed by several studies performed in the following different test models: in carrageenan and PGE2-induced hind paw edema, TPA-induced mouse ear edema in vivo, acetic acid-induced capillary permeability test, inhibitory activity test on TNF-α and IL-1*β* production as proinflammatory cytokines, and NF-κB nuclear translocation in THP-1 macrophages in vitro [17,20,23].

The current study focused on the anti-inflammatory activity of *G. tomentosa* and isolation of the active component responsible for its activity. The plant species were evaluated for their activities in the current study using human red blood cell membrane-stabilizing activity to determine the most active species. *G. tomentosa* was detected to be the most active species among all tested species. According to the activity-guided approach, liquid/liquid fractionation was applied to the methanolic extract to obtain *n*-hexane, dichloromethane, ethyl acetate, and the remaining parts, respectively. Fractionation of the ethyl acetate layer on the silica gel column and following further chromatographic techniques resulted in the isolation of sixteen different structures from different classes of secondary metabolites, including flavonoids, phenolic acids, lignan, and coumarin. The structural identity of the compounds was established by using spectral analysis, including ^1^H and ^13^C, as well as 2D nuclear magnetic resonance (NMR) and mass spectral (MS) techniques, as necessary. All isolated compounds were evaluated for their potential anti-inflammatory activities by using the same test model. Physicochemical properties and pharmacokinetic characteristics, as well as pathways and targets associated with anti-inflammatory effects of these compounds, were predicted and evaluated using in silico models.

## 2. Results

### 2.1. In Vitro Study Results

The current study was designed to evaluate the anti-inflammatory potential of plants, which are used in folk medicines for inflammation-related diseases, and to isolate the responsible compound. *Podospermum canum* (Syn. *S. cana* (C.A. Meyer) Hoffm. var. *jacquiniana* (W. Koch) Chamb.), *Gelasia cinerea* (Boiss.) Zaika, Sukhor. & N.Kilian (Syn. *S. cinerea* Boiss., *Pseudopodospermum szowitzii* (DC.) Kuth. (Syn. *S. mollis* Bieb. ssp. *szowitzii* (DC.) Chamb.), *G. latifolia* (Fisch. & C.A.Mey.) Zaika, Sukhor.& N.Kilian (Syn. *S. latifolia* (Fisch. and Mey.) DC., *S. parviflora* Jacq., and *S. tomentosa* L., which is known as *Gelasia tomentosa* (L.) Zaika, Sukhor. & N.Kilian anymore were evaluated for their protective activities on the human red blood cell membrane against heat-induced hemolysis to prove anti-inflammatory potential and determine the highest activity. Among the extracts tested, *G. tomentosa* demonstrated the most remarkable protective activity (IC_50_ = 5.71 mg/mL), followed by *Podospermum canum* (IC_50_ = 5.81 mg/mL) and *G. latifolia* (IC_50_ = 5.85 mg/mL). However, the activity of acetylsalicylic acid (ASA), positive control, was significantly higher than these extracts. All results were displayed in Table 1.

To identify bioactive compounds, a methanolic extract of the aerial parts of *G. tomentosa* was subjected to liquid–liquid partitioning, yielding n-hexane, dichloromethane, ethyl acetate, and residual methanol–water phases. Each phase demonstrated significant protective effects against heat-induced hemolysis in human red blood cell membranes, with responses correlating with concentration. Notably, the ethyl acetate and dichloromethane phases exhibited the strongest protective activities, both showing IC_50_ values of 0.98 mg/mL, as indicated in Table 2.

The ethyl acetate and dichloromethane fractions were selected for further investigation. The dichloromethane phase underwent silica gel column chromatography using a gradient of dichloromethane and methanol to achieve 103 fractions. These fractions were subsequently grouped based on their TLC profiles, labeled STC1-9, and assessed for anti-inflammatory properties. The fractions derived from the dichloromethane phase displayed pronounced protective effects compared to the control, with STC4 emerging as the most potent, followed closely by STC3, as documented in Table 3, exhibiting IC_50_ values of 0.39 and 0.41 mg/mL, respectively.

The ethyl acetate phase was also separated by column chromatography to yield forty-seven fractions that were combined according to their phytochemical profiles as STAE1-9. The combined fractions were reevaluated for their in vitro potential anti-inflammatory properties. Each fraction demonstrated a protective effect on human red blood cell membranes against heat-induced hemolysis, as summarized in Table 4. Notably, STAE4 demonstrated the most significant membrane stabilization, followed closely by STAE9, with IC_50_ values of 0.16 mg/mL and 0.21 mg/mL, respectively. Both fractions demonstrated superior activity compared to the positive control. The STAE4 fraction was identified as particularly significant and underwent further chromatographic purification via semi-preparative HPLC on an RP-C18 column, utilizing an ACN/water gradient elution. A total of sixteen distinct compounds were successfully isolated from the STAE4 fraction, which includes a range of chemical classes: flavonoids, caffeoylquinic acids, lignans, and megastigmane glycosides.

**Figure 1:**
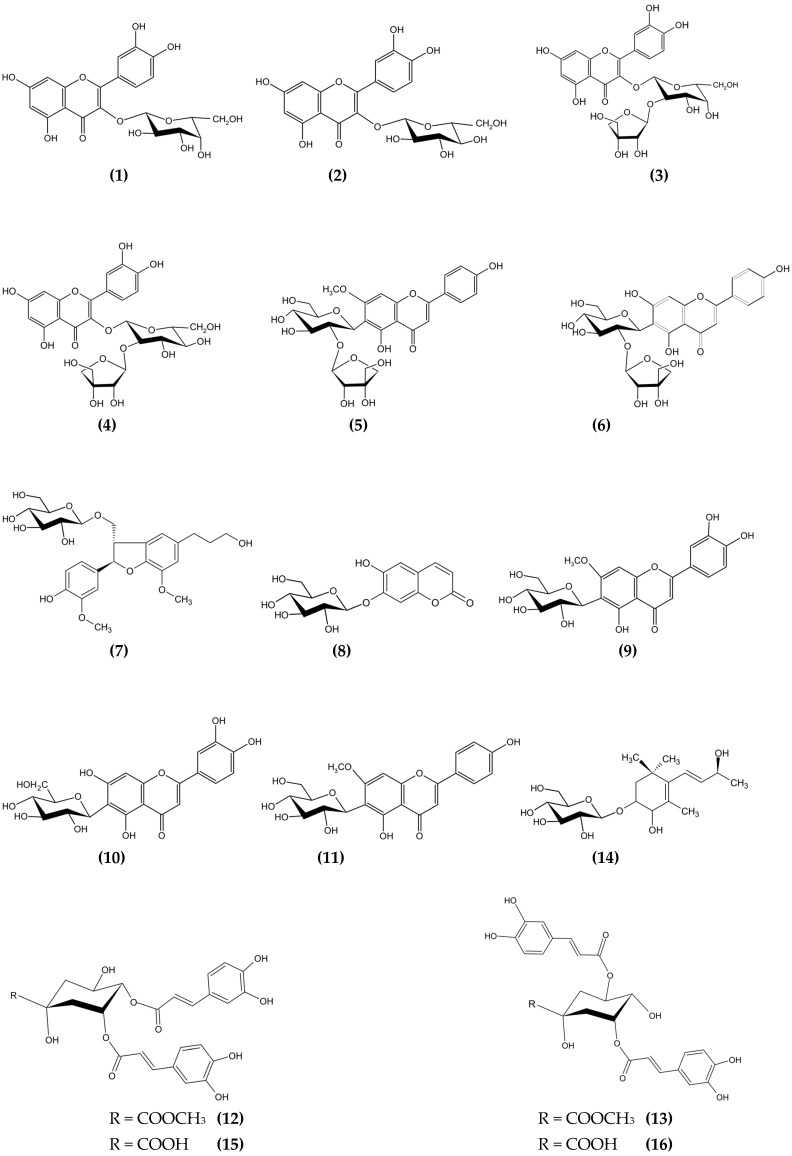
Isolated compounds (**1**–**16**) from *G. tomentosa* aerial parts ethyl acetate phase.

Various spectral analysis methods (NMR (^1^H, ^13^C, 2D NMR), MS) were used to establish the structure of all isolated compounds. Compounds **1** (6.8 mg), **2** (10 mg), **9** (5.6 mg), **10** (4.8 mg), and **11** (8.9 mg) were obtained as yellow powders, and molecular weight as well as ^1^H, ^13^C-NMR data comparison by the data from the literature allowed to the structure elucidation as hyperoside, isoquercitrin, 7-*O*-methyl-isoorientin, isoorientin, and swertisin, respectively [20,24,25]. Compounds **3** (3.8 mg), **4** (6.5 mg), **5** (4.3 mg), and **6** (11.2 mg) were obtained by repeated purification procedures using semi-prep HPLC analysis. Both compounds **3** and **4** were determined to be quercetin derivatives and very similar to each other, even with molecular weights that were measured as *m*/*z* 619.86 [M + Na]^+^ and 619.93 [M + Na]^+^ for each. Quercetin moiety was detected easily from the ^1^H and ^13^C NMR spectra, while the sugar part was partially complicated. The presence of characteristic signals determined the apiosyl moiety, and the connections were detected by the observed HMBC correlations between and the shifted signals of H-1‴ (δ 5.35 (1H, *J* = 1.6) and δ 5.37 (1H, *J* = 1.6) ppm) and C-2″ (δ 76.62 and δ 77.55 ppm) for compound 3 and 4, respectively. Compound **3** and **4** structures were elucidated as quercetin 3-*O*-*β*-apiofuranosyl-(1→2)-*β*-galactopyranoside and quercetin 3-*O*-*β*-apiofuranosyl-(1→2)-*β*-glucopyranoside, respectively [26,27]. Compounds **5** and **6** were also obtained as yellow powder, and the compounds’ UV spectra indicated a flavonoid structure. The measured molecular weight of compound **5** as *m*/*z* 566.18 [M + H]^+^ suggested the presence of a flavonoid aglycone and two monosaccharide units. Its ^1^H-NMR and MS results proved the structure of this as the apigenin 6-*C*-[*β*-apiofuranosyl-(1→2)]-*β*-glucopyranoside. ESI-MS of compound **6** (10 mg) displayed *m*/*z* 578.24 [M − H]^−^ as molecular weight. Apigenin structure was proved by the existence of δ 7.86 (2H, d, *J* = 8.8, H-2′ and H-6′), δ 6.92 (2H, d, *J* = 8.4, H-3′ and H-5′), δ 6.61 (1H, s, H-6/8), δ 6.51 (1H, s, H-3) proton signals by ^1^H NMR spectra. The absence of the proton signal at H-6 suggests the presence of a C-glycoside of apigenin, as confirmed by the anomeric proton (δ 4.88 ppm, H-1″) and carbon signals (C-1″ at 73.33/72.88, C-1‴ at 111.01/110.92) in the NMR spectra. HMBC correlations indicated a C-6 connection with δ 4.88 ppm (H-1″) and carbon signals at 167.24/165.32 (C-7), 162.02/161.01 (C-5), and 110.69/110.59 (C-6). Additionally, the signals at δ 3.93/3.92 ppm (OCH_3_) and δ 57.01/56.81 ppm supported -OCH_3_ substitutions at the C-7 position, confirmed by HMBC correlation. Consequently, compound **6** was identified as 7-methoxyapigenin-6-*C*-[*β*-apiofuranosyl-(1→2)]-*β*-glucopyranoside [28,29]. Dihydrodehydrodiconiferyl alcohol-4-*O*-*β*-glucopyranoside (compound **7**, 14.2 mg) was identified through ESI-MS (*m*/*z* 546.13 [M + Na]^+^), ^1^H-, and ^13^C-NMR, including 2D-NMR. The ^1^H-NMR spectrum showed methylene signals at δ 1.80 ppm (H-8′), δ 2.62 ppm (H-7′), δ 3.56 ppm (H-9′), and δ 3.74–3.84 ppm (H-9). Methoxy substitutions appeared at δ 3.82 ppm and δ 3.85 ppm for protons, with respective carbon signals at δ 56.77 ppm and δ 56.70 ppm. Twelve carbon signals (δ 111.13–150.95 ppm) indicated two different benzene cycles: one trisubstituted and another indicated by five aromatic carbon signals (e.g., δ 7.13, H-5). The furan skeleton was suggested by proton signals (δ 5.55, H-7; δ 3.46, H-8) and confirmed by HMBC correlations. The glucose structure was identified with specific proton and carbon signals (e.g., δ 4.8, H-1″; δ 102.77, C-1″). All data matched the literature, allowing elucidation of the compound’s structure [30]. Compound **8** (4.1 mg), obtained as white crystals with the molecular weight measured as *m*/*z* 339.91 [M − H]^−^, *m*/*z* 341.35 [M + H]^+^by ESI-MS, gave very characteristic signals as δ 7.82 (1H, d, *J* = 10.0 Hz, H-4), δ 7.20 (1H, s, H-8), δ 7.03 (1H, s, H-5), δ 6.28 (1H, d, *J* = 9.6 Hz, H-3) for simple coumarin structure on ^1^H-NMR spectra, confirming the compound **8** as cichoriin, which is one of the most common simple coumarin glycosides distributed in the Asteraceae family [31]. Compounds **12** (4.4 mg), **13** (10 mg), **15** (10 mg), and **16** (4.5 mg) were purified by preparative TLC on reverse-phase TLC plates (Merck 5559, Darmstadt, Germany) using the MeOH:water (4:6) solvent system. The UV spectra of compounds **12**, **13**, **15**, and **16** indicated that they were caffeoylquinic acid derivatives. The molecular weights of compounds **15** and **16** were measured as *m*/*z* 516.10 [M − H]^−^, indicating they are dicaffeoylquinic acid derivatives. NMR analysis identified them as 3,5-*O*-dicaffeoylquinic acid and 4,5-*O*-dicaffeoylquinic acid. Other compounds with *m*/*z* 531.82 [M − H]^−^ and *m*/*z* 531.89 [M − H]^−^ were identified as methyl esters of these dicaffeoylquinic acids [32,33,34]. The white solid residue obtained as compound **14** (4.6 mg) with molecular weight *m*/*z* 411.9 [M + Na]^+^ and ^1^H, ^13^C NMR data displayed that the compound was Staphylionoside E, a megastigmane glycoside derivative [35]. The chemical structures of the all isolated compounds are displayed in Figure 1.

According to the in vitro anti-inflammatory activity results, 4,5-*O*-dicaffeoylquinic acid (**16**) exhibited the highest membrane-stabilizing activity, followed by 7-methoxyapigenin 6-*C*-*β*-apiofuranosyl-(1→2)-*β*-glucopyranoside (**5**) (Table 5). 3,5-*O*-dicaffeoylquinic acid (**15**) and its methyl ester (**12**) were also determined to have remarkable anti-inflammatory activity with 0.87 and 0.83 IC_50_ values, respectively. Hyperoside (**1**), 7-*O*-methylisoorientin (**9**), quercetin 3-*O*-*β*-apiofuranosyl-(1→2)-*β*-galactopyranoside (**3**), and isoorientin (**10**) displayed membrane-stabilizing activity with IC_50_ values that measured under 1 mg/mL, while the remaining compounds were not detected to have significant activity. The compounds **1**, **3**, **5**, **9**, **10**, **12**, **13**, and **15**, **16** isolated from *G. tomentosa* aerial parts exhibited higher putative anti-inflammatory activities than the total methanolic extract and ethyl acetate phase, while their activities were detected to be lower than the several fractions of ethyl acetate and dichloromethane phases, especially the most active fraction of ethyl acetate (STAE4).

### 2.2. In Silico Study Results

Physicochemical and absorption, distribution, metabolism and excretion (ADME)/pharmacokinetic profiles and anti-inflammatory potentials of the isolated compounds through different pathways and targets were evaluated using in silico models implemented in ADMETlab 3.0 [36,37]. The names, Simplified Molecular Input Line Entry System (SMILES), molecular formulas, molecular weights, and 2D structures of the isolated compounds (*n* = 16) are presented in the Supplementary Table S1.

#### 2.2.1. In Silico Predictions of Physicochemical and ADME/Pharmacokinetic Properties

**Figure 2:**
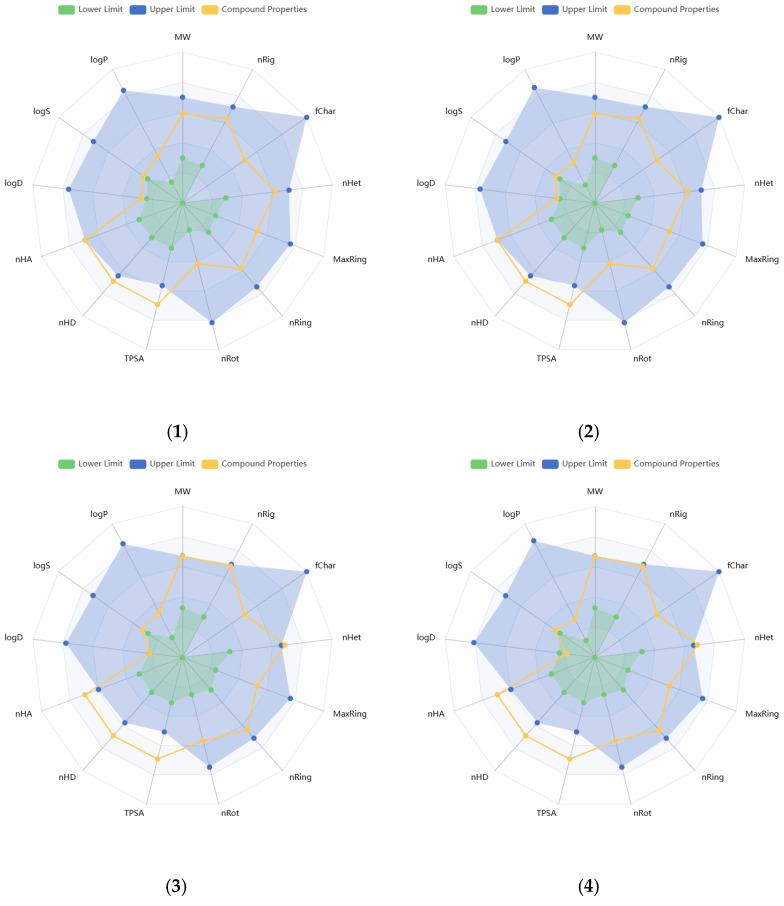

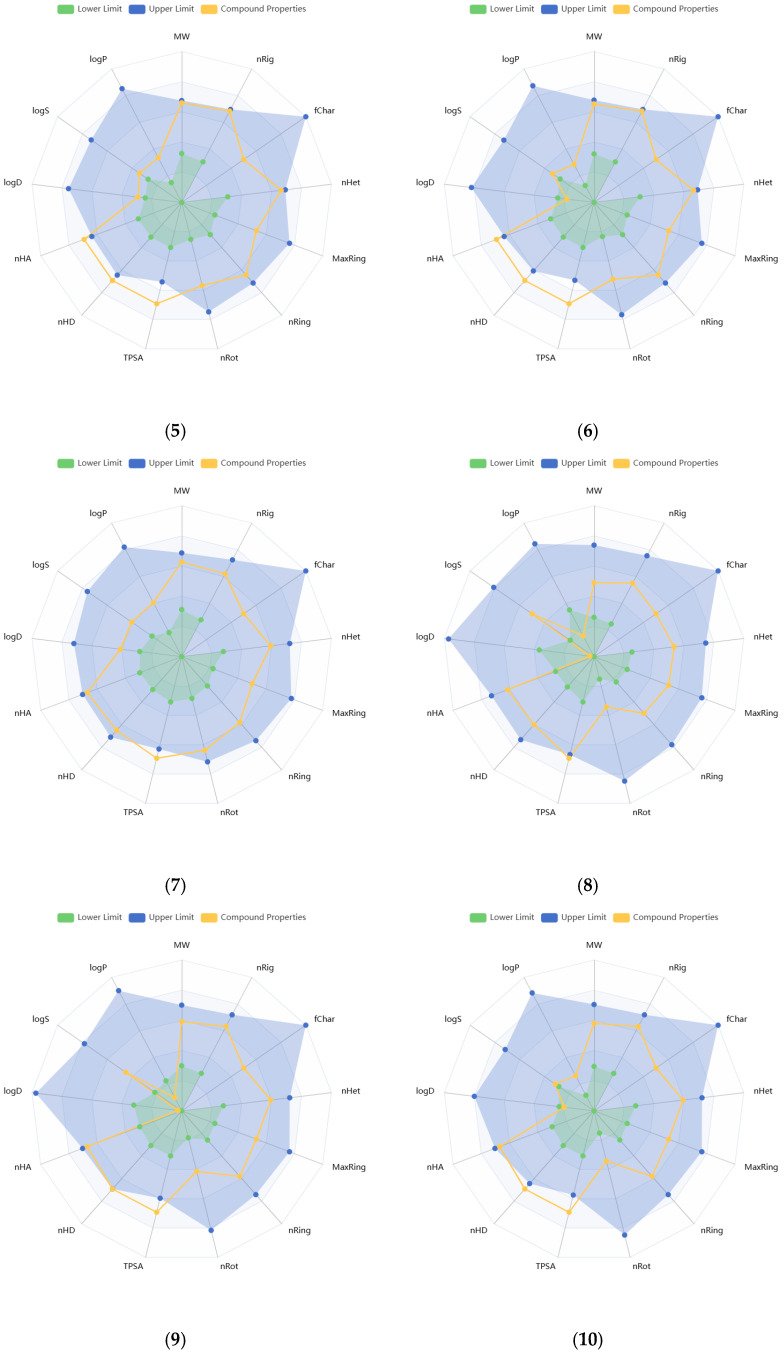

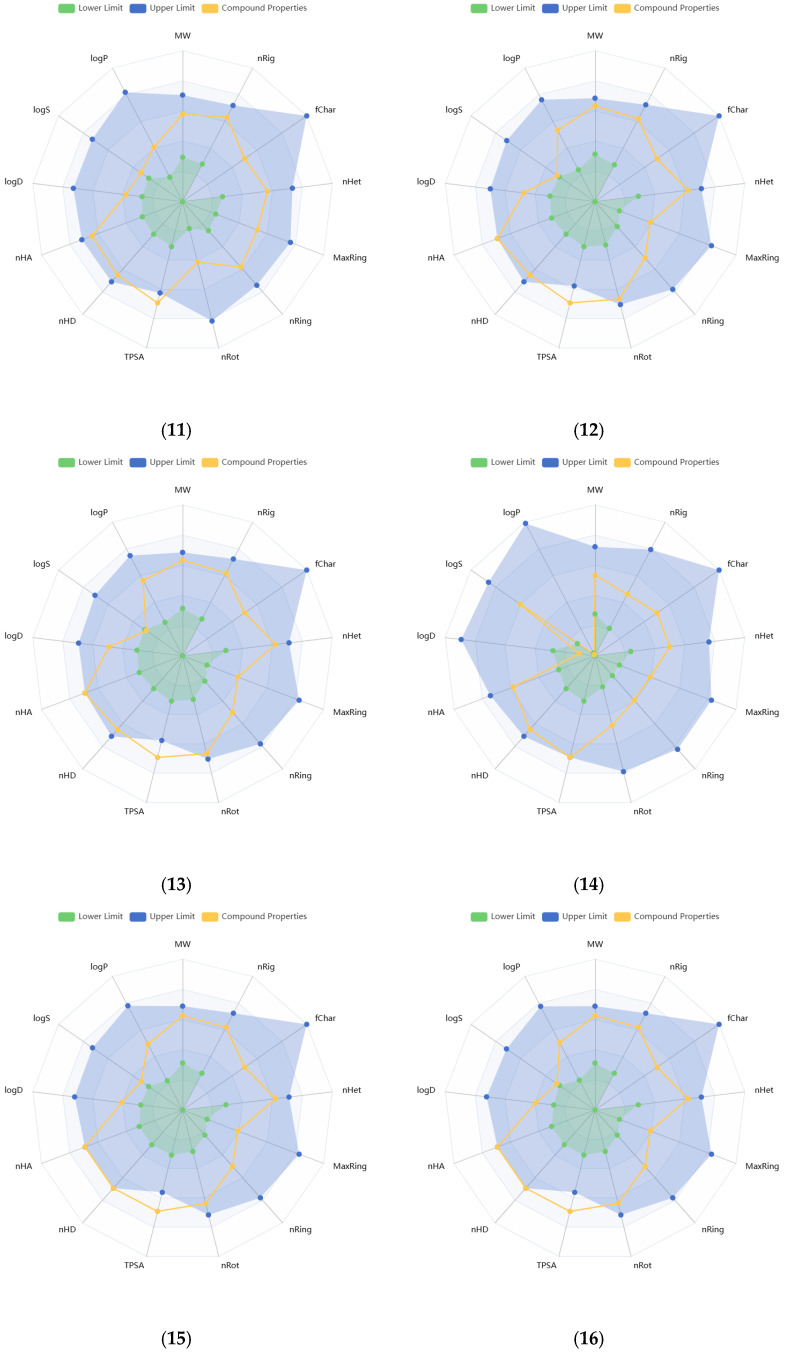
Radar plots of oral bioavailability and physicochemical properties of the isolated compounds (**1**–**16**).

Physicochemical properties of the compounds were predicted, and the resulting data are presented in Supplementary Tables S2 and S3. Molecular weights of the compounds range from 340.28 to 596.49 g/mol. The number of hydrogen bond acceptors (Hacc) (≤12) for compounds **1**, **2**, **7**–**11**, and **14**, and the number of hydrogen bond donors (Hdon) (≤7) for compounds **8**, **9**, **11**–**16** were found to be optimal, based on the drug-like soft rule. The number of rotatable bonds (*n* < 11), rings (*n* < 6), atoms in the biggest ring (MR < 18), and rigid bonds (*n* < 30) was determined to be optimal for all compounds regarding the drug-like soft rule. Compounds **12** and **15** satisfied the Lead-Like Soft rule with a level of two in stereo centers (SCs). The SCs of the other compounds were found to be between 4 and 8. The topological polar surface area (TPSA) of almost all compounds was estimated to be higher than 140 Å^2^. The logS value of compound **14** was estimated to be −1.116 log mol/L, that of **8** was −1.746 log mol/L, and that of **9** was −2.141 log mol/L. The logP values of these same three compounds were found as −0.034, −1.178, and −0.555 log mol/L, respectively. These values indicate higher hydrophilicity and solubility. The logS values of the other compounds were found to be lower than −2.5, while logP values were between 0 and 2. Compound **14** was predicted to have the lowest melting and boiling points (111.498 and 263.28 °C, respectively), while **4** was estimated to have the highest ones (287.848 and 398.743 °C, respectively). Figure 2 illustrates the oral bioavailability and physicochemical property radar chart of the solated compounds.

Medicinal chemistry properties of the compounds were predicted, and the corresponding results are displayed in the Supplementary Table S4. None of the compounds has a quantitative estimate of drug-likeness (QED) value higher than 0.67. The highest QED value was estimated at 0.429, and it belongs to compound **8**. SAScores were found “easy” for all compounds. Graph attention-based assessment of synthetic accessibility (GASA) was estimated to be easy for 56.25% of the compounds. The maximum estimated fraction sp3 (Fsp3) value was 0.789, observed for **14**. Fsp3 of **3**–**7** were also found to be higher than 0.42. The medicinal chemistry 2018 (MCE-18) values were estimated to exceed 45 for all compounds. The MCE-18 values of 87.5% of the compounds were even greater than 78. Natural product-likeness scores of the compounds range from 1.294 to 2.607. Lipinski’s Rule of Five indicates MW ≤ 500, logP ≤ 5, Hacc ≤ 10, Hdon ≤ 5. Pfizer’s Rule refers to logP > 3 and TPSA < 75, while GSK’s Rule indicates MW ≤ 400. Golden Triangle Rule supports logP ≤ 4, 200 ≤ MW ≤ 500, and −2 ≤ logD ≤ 5 [38,39]. Compounds **8** and **14** obeyed the Lipinski’s Rule of Five, Pfizer’s Rule, GSK’s Rule, and the Golden Triangle Rule. However, swertisin (**11**) satisfied three of these rules, excluding GSK. A total of 62.5% of the compounds were estimated to have one PAINS alert.

ADME/pharmacokinetic profiles of the compounds were determined using several models, with the outputs summarized in the Supplementary Tables S5 (Absorbtion and Distribution: A-D) and S6 (Metabolism and Elimination: M-E).

Predicted Caco-2 permeability values of all compounds were lower than −5.15 log cm/s, suggesting lower permeability. Predicted Madin−Darby canine kidney cells (MDCK) and parallel artificial membrane permeability values also indicated low permeability. None of the compounds was predicted as a P-glycoprotein (Pgp) inhibitor. Compounds **6** and **14** exhibited prediction scores exceeding 0.5 for Pgp substrate status, with values of 0.559 and 0.838, respectively. The probability of being a Pgp substrate for the other compounds was found to be less than 0.35. The probability of absorption of compounds from the human intestine was estimated to range from 0.1 to 0.956. The maximum prediction scores were 0.956 and 0.952, corresponding to compounds **5** and **3**, respectively. The model description defines bioavailability values as Category 1 F20% + (bioavailability < 20%) and Category 0 F20%- (bioavailability ≥ 20%). Compound **7** yielded the lowest F20% prediction value of 0.227. Similarly, the prediction scores of **2**, **11**, **9**, and **14** were all below the 0.5 threshold. The predicted F30% and F50% for nearly all compounds approached one, suggesting poor bioavailability.

All compounds can be considered to have proper plasma protein binding (PPB), with predicted values below 90%. The fraction unbound in plasma (Fu) for the compounds was estimated to be 12.7% to 41.75%, with the highest value observed for compound **8**. The volumes of distribution at steady state (VDss) of all compounds were within the acceptable range. The penetration of compounds through the blood–brain barrier was estimated to be negligible or very low. Half of the compounds were predicted to be MRP1 inhibitors with different probability scores. The maximum prediction value, 0.945, was observed for compound **8**. The majority of the compounds were predicted to inhibit organic anion-transporting polypeptide (OATP) 1B1, while all were anticipated to inhibit 1B3.

None of the compounds was predicted to be inhibitors or substrates of CYP1A2, CYP2C9, CYP2C19, and CYP2D6. Approximately 90% of the compounds were estimated to inhibit CYP2C8, whereas 25% were expected to inhibit CYP3A4. The human liver microsomal stability of the compounds was determined as low. The plasma clearance (CLplasma) of the compounds ranged from low to moderate. The half-life (T_1/2_) of them was determined between 2 and 4.5 h, which can be considered moderate.

#### 2.2.2. In Silico Prediction of Anti-Inflammatory Activity and Target Pathways

The anti-inflammatory activity was examined by evaluating their potential as anti-inflammatory agents, immunostimulants, immunomodulators, and enhancers of tumor protein (TP) 53 expression, as well as inhibitors of TNF expression. The results are shown in Table 6. The names of the compounds indicated by numbers in Table 6, Table 7 and Table 8 are as follows: hyperoside (**1**), isoquercetin (**2**), quercetin 3-*O*-*β*-apiofuranosyl-(1→2)-*β*-galactopyranoside (**3**), quercetin 3-*O*-*β*-apiofuranosyl-(1→2)-*β*-glucopyranoside (**4**), 7-methoxyapigenin-6-*C*-*β*-apiofuranosyl-(1→2)-*β*-glucopyranoside (**5**), apigenin-6-*C*-*β*-apiofuranosyl-(1→2)-*β*-glucopyranoside (**6**), dihydrodehydrodiconiferyl-alcohol-4-*O*-*β*-glucopyranoside (**7**), cichoriin (**8**), 7-*O*-methylisoorientin (**9**), isoorientin (**10**), swertisin (**11**), 3,5-*O*-dicaffeoylquinic acid methyl ester (**12**), 4,5-*O*-dicaffeoylquinic acid methyl ester (**13**), staphylinioside E (**14**), 3,5-*O*-dicaffeoylquinic acid (**15**), and 4,5-*O*-dicaffeoylquinic acid (**16**)**.** All isolated compounds (*n* = 16) were predicted to possess anti-inflammatory effects regarding Pa and Pi values. Pa values ranged from 0.321 to 0.770. The highest Pa values for anti-inflammatory activity, which were higher than 0.7, belonged to compounds **3**, **4**, **7**, **1**, **2**, **8**, **13**, and **12**, respectively. All isolated compounds (*n* = 16) were anticipated to be TP53 expression enhancers with a Pa value ranging from 0.614 to 0.959. Isoorientin (**10**) exhibits the highest Pa value for enhancing TP53 expression, followed by hyperoside (**1**) and isoquercetin (**2**), respectively. Four compounds (**1**, **2**, **8**, and **14**) were estimated to have immunomodulatory activity; however, their Pa values were found to be lower than 0.4. Eight of the compounds proved to be immunostimulants with a Pa value greater than 0.5. The highest Pa value was observed for compounds **3**, **4**. Eight compounds (**1**–**8**) were not predicted to inhibit TNF, while **12**, **13** and **15**, **16** have Pa values slightly above 0.5.

The antagonistic effects of the compounds on several ILs, an essential cytokine family associated with inflammation, were also investigated. The results were displayed in Table 7. None of the compounds provided a Pa value higher than 0.5 to antagonize any of the ILs tested. This indicates that none of the compounds were strongly likely found to be IL antagonists. These prediction results support previous studies [20].

The effects of the compounds on the cell membrane were also evaluated in silico to support in vitro data, as shown in Table 8. All isolated compounds showed a high probability of being membrane integrity agonists, with activity probability values ranging from 0.825 to 0.989. Compounds **1** and **2** demonstrated the highest Pa value of 0.989 (Pi = 0.001), followed closely by compounds **3** and **4** with Pa values of 0.970 (Pi = 0.002). Notably, all isolated compounds indicated significant potential to inhibit membrane permeability, with Pa values ranging from 0.680 to 0.981. Compounds **1** and **2** were particularly noteworthy, being categorized as the most potent membrane permeability inhibitors (Pa = 0.981, Pi = 0.001) and also displaying the highest efficacy as cardioprotective and hemostatic agents with Pa values of 0.984 and 0.987, respectively.

**Figure 3:**
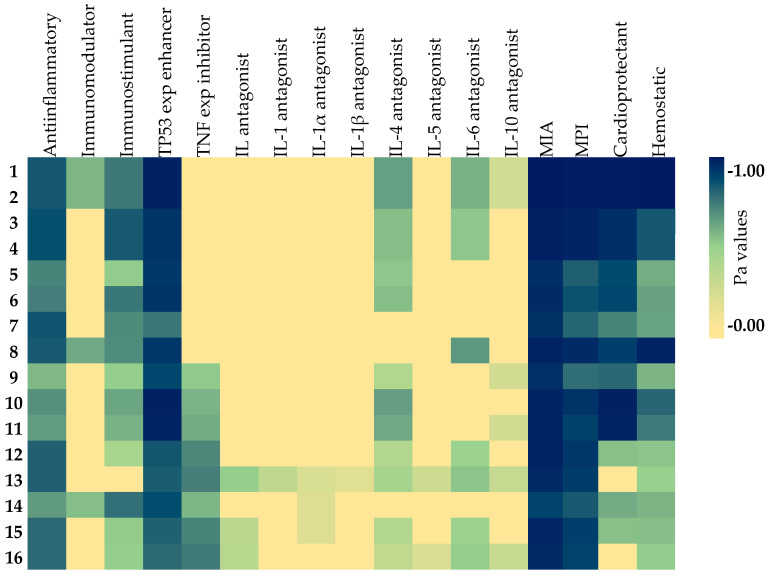
Heatmap of the predicted anti-inflammatory and inflammation-related activities of the isolated compounds. As the color darkens, the activity prediction percentage increases. Exp: expression; IL: interleukin; MIA: membrane integrity agonist; MPI: membrane permeability inhibitor; TNF: tumor necrosis factor; TP: tumor protein.

From a broader perspective, in vitro and in silico analysis results suggest that these isolated compounds can attenuate inflammation in blood cell membranes, thereby exerting their anti-inflammatory effects on the cardiovascular system indirectly. The heatmap in Figure 3 shows the predictions of anti-inflammatory and inflammation-related activities generated by in silico models.

## 3. Discussion

The findings from the current investigation indicate that the most active fraction of *G. tomentosa* aerial parts is rich in flavonoid glycosides and dicaffeoylquinic acid derivatives. Notably, among the isolated chemical entities, the following nine have been identified as flavonoid glycosides: hyperoside (**1**), isoquercetin (**2**), quercetin-3-*O*-*β*-apiofuranosyl-(1→2)-*β*-galactopyranoside (**3**), quercetin 3-*O*-*β*-apiofuranosyl-(1→2)-*β*-glucopyranoside (**4**), 7-methoxyapigenin 6-*C*-*β*-apiofuranosyl-(1→2)-*β*-glucopyranoside (**5**), apigenin 6-*C*-*β*-apiofuranosyl-(1→2)-*β*-glucopyranoside (**6**), 7-*O*-methylisoorientin (**9**), isoorientin (**10**), swertisin (**11**); four of them caffeoylquinic acid derivatives 3,5-*O*-dicaffeoylquinic acid (**15**) and 4,5-*O*-dicaffeoylquinic acid (**16**) and their methyl esters (**12**, **13**). Furthermore, one coumarin glycoside as cichoriin (**8**), one lignan glycoside, dihydrodehydrodiconiferyl alcohol 4-*O*-*β*-glucopyranoside (**7**), and one megastigmane glycoside as staphylionoside E (**14**) have already been isolated. Previous phytochemical investigations on *G. tomentosa* have successfully revealed the isolation of several compounds from its root, including chlorogenic acid, (±)-scorzophtalide, (±)-scorzotomentosin, (−)-scorzotomentosin, (−)-scorzotomentosin-4′-*O*-*β*-glucoside, (±)-hydrangenol, (−)-hydrangenol-4′-*O*-*β*-glucoside, scorzoerzincanin, (±)-hydramacrophyllol A, and (±)-hydramacrophyllol B [40]. Additionally, a study on the sterol profile of *G. tomentosa* identified *β*-sitosterol and stigmasterol 3-*O*-*β*-glycoside [41]. Regarding aerial parts, an n-hexane extract revealed the presence of lupeol, lupeol acetate, and taraxasteryl acetate, while the root contains lupeol acetate, lupeol, taraxasteryl acetate, 3*β*-hydroxy-fern-7-en-6-one-acetate, α-amyrin, and olean-12-en-11-one-3-acetyl in n-hexane extract [42]. In terms of aqueous methanolic extracts of the aerial parts, HPLC analysis confirmed the presence of chlorogenic acid, hyperoside, and luteolin 7-*O*-*β*-glucoside [17]. Notably, chlorogenic acid emerged as a prominent component in extracts obtained from both the aerial parts and roots [43].

*G. tomentosa*, which is formerly classified under *Scorzonera* genus, belonging to the Scrozonerinae subtribe of the Cichorieae, is chemically characterized by the existence of triterpenes, sesquiterpenes, sesquiterpene lactones, flavonoids, dihydroisocoumarines, bibenzyl derivatives, lignans, quinic, and caffeic acid derivatives, as well as dihydrostilbenes. Regarding flavonoid accumulation, however, the genus plants have not yet been adequately investigated for their flavonoid profiles. The Scorzonerinae subtribe has been described by the presence of mainly C-glycosides of apigenin and luteolin, followed by luteolin *O*-glycosides, as well as quercetin derivatives, which were determined to be the fourth dominant group of flavonoids. Current research has resulted in the isolation of the quercetin-*O*-glycosides mainly and C-glycosides of both luteolin and apigenin. On the other hand, four different dicaffeoylquinic acid derivatives were isolated as major components of the *G. tomentosa* aerial parts ethyl acetate phase. Previously, it was reported that different types of caffeic acid derivatives occur in the *Scorzonera* genus, and unidentified structures have been isolated previously. The Scorzonerinae subtribe has been described by the occurrence of caffeic acid derivatives, mainly mono- and di-caffeoyl quinic acid types, while caffeoyl tartaric acid derivatives, which are known to be typical for some subtribes of the Cichorieae, were reported to be absent in the taxa of the Scorzonerinae [44,45]. Our study results were supported by the findings from the isolation of four different types of dicaffeoylquinic acid derivatives as major constituents of *G. tomentosa*.

Flavonoids display anti-inflammatory activities through several mechanisms. For example, the inhibitory activities of regulatory enzymes and transcription factors, which are the most important ones for flavonoids, allow the modulation of mediators in the inflammation area and result in decreased signal transduction as well as cell activation. Due to their phenolic structures, flavonoids also display amazing antioxidant activities in different ways and suppress oxidative stress, which occurs during the inflammation process and is harmful to cell functions. In addition, flavonoids affect the metabolism of arachidonic acid which is released from phospholipids by inflammation and triggers successive processes for the biosynthesis of prostaglandins, thromboxanes, leukotrienes, and other inflammatory mediators, leading to a decreased production of proinflammatory mediators. Furthermore, the modulation activities of flavonoids on gene expression by affecting transcription factors induced a decrease in proinflammatory gene transcription. Another property of flavonoids that is helpful for the inflammatory process is that it is reported that their regulatory activities on cell activation, maturation, signaling transduction, cytokine production, and secretory processes in immune cells, which cause maturation of cell stages, decrease cell proliferation, and decrease in release of proinflammatory cytokines play a significant role in the anti-inflammatory process [46]. 7-methoxyapigenin-6-*C*-*β*-apiofuranosyl-(1→2)-*β*-glucopyranoside (**5**), hyperoside (**1**), quercetin 3-*O*-*β*-apiofuranosyl-(1→2)-*β*-galactopyranoside (**3**), 7-*O*-methylisoorientin (**9**), and isoorientin (**10**), among the isolated flavonoids, exhibit notable anti-inflammatory activity, but the activities were not higher than ASA. On the other hand, all isolated caffeoylquinic acid derivatives were found to have red blood cell membrane-stabilizing activity in varying degrees, while the 4,5-*O*-dicaffeoylquinic acid was detected as the highest one, which was almost the same as the two-fold IC_50_ value of the ASA. Several researchers have also reported the anti-inflammatory activity of the dicaffeoylquinic acid derivatives. Previous studies have reported that pretreatment of mice by oral administration with 3,5-*O*-dicaffeoylquinic acid and 4,5-*O*-dicaffeoylquinic acid displayed moderate inhibition in carrageenan-induced edema after 3h of induction and a significant decrease in the levels of TNF-α moderately and IL-1*β* [47]. Another study has revealed that the dicaffeoylquinic acid derivatives regulate the MAPKs’ signaling pathway through the modulation of signaling proteins such as ERK, JNK, and p38, significantly decreasing the phosphorylation of the mentioned proteins in RAW264.7 macrophage cells [48].

Current research indicates that fractions display enhanced biological activity compared to their isolated counterparts, hinting at potential synergistic or additive interactions among their constituents. Notably, several studies have documented synergistic effects among flavonoids, where specific combinations have led to a significant reduction in inflammatory mediators, including nitric oxide, prostaglandin E_2_, and TNF-α in macrophage models [49,50]. Citing these established interactions could provide valuable context for interpreting the present findings and elucidating the mechanisms behind the superior efficacy of fractions compared to isolated pure compounds.

The heat-induced hemolysis assay is widely recognized as a valuable preliminary model for assessing membrane-stabilizing properties potentially associated with anti-inflammatory effects. The relevance of this assay lies in its ability to protect erythrocytes from thermal lysis, which can be analogous to the stabilization of lysosomal membranes. Such stabilization has implications for preventing the release of proinflammatory mediators during inflammatory responses [51,52] A brief elucidation of this mechanistic relationship would strengthen the justification for utilizing this model in the study. Although the HRBC assay is a useful screening tool, it does have its limitations. The lack of nuclei and immune response pathways in erythrocytes restricts its relevance to the physiological context of inflammatory cells Additionally, the assay fails to quantify key inflammatory mediators such as prostaglandins and cytokines, and the presence of membrane-active compounds may lead to false positive results. Moreover, significant variability in donor blood can affect the outcomes associated with membrane fragility [53,54]. Consequently, results from HRBC membrane-stabilization assays should be regarded as preliminary indicators of potential anti-inflammatory activity. A thorough understanding of the underlying mechanisms requires further corroboration through cellular, biochemical, and in vivo models. Additional studies on isolated compounds will provide insights into the precise mechanisms of their anti-inflammatory effects.

The isolated compounds were assessed using in silico methods to determine their physicochemical properties, pharmacokinetic behavior, and ADME profiles, to evaluate their suitability as anti-inflammatory drug candidates. ADMETLab 3.0 has explained Lipinski’s Rule of Five, Pfizer’s Rule, GSK’s Rule, and the Golden Triangle Rule as follows: Lipinski’s Rule MW ≤ 500, logP ≤ 5, Hacc ≤ 10, Hdon ≤ 5; GSK’s Rule MW ≤ 400, logP ≤ 4; Golden Triangle Rule 200 ≤ MW ≤ 500, −2 ≤ logD ≤ 5. According to Lipinski’s Rule, deviations in two physicochemical parameters suggest a potential for poor absorption or permeability, whereas a single violation is generally considered acceptable. Pfizer’s Rule indicates that compounds with a high logP (>3) and low TPSA (<75) are likely to be toxic. MW of cichoriin (**8**) is 340.28 g/mol, logP is −1.178 log mol/L, number of Hacc is 9, number of Hdon is 5, TPSA is 149.82 Å, logD is −0.115 log mol/L. MW of staphylinioside E (**14**) is 388.45 g/mol, logP is −0.034 log mol/L, number of Hacc is 8, number of Hdon is 6, TPSA is 139.84 Å, logD is 0.431 log mol/L. Within these physicochemical parameters, cichoriin (**8**) and staphylinioside E (**14**) mostly satisfy Lipinski’s Rule of Five, GSK’s Rule, and the Golden Triangle Rule, suggesting more favorable ADME profiles, and they are not likely to be toxic according to Pfizer’s Rule. The numbers of Hacc and Hdon are crucial for a compound’s polarity and solubility, and can affect passive diffusion across cell membranes, a fundamental process in absorption and distribution. A moderate number of Hdon (≤7) maintains sufficient resolution without impeding permeability. Therefore, they are used as key molecular descriptors to predict the oral bioavailability of drug candidate molecules [55,56]. The number of rotatable bonds is widely accepted as the most common descriptor of flexibility, which influences oral bioavailability. The unrestricted rotation of atoms around single bonds enables the molecule to adopt multiple conformations, and so, molecular flexibility increases with the number of such bonds. In general, for new drug candidate molecules, ≤10 rotatable bonds are considered more suitable for both target binding and membrane permeability [57,58]. An excessive number of rings can make the molecule too hydrophobic, reduce its solubility in water, and impair its metabolic stability. The presence of more than three aromatic rings in a molecule is associated with reduced developability of the compound. A greater number of atoms in the largest ring (MaxRing) makes it more difficult for the molecule to pass through the cell membrane by passive diffusion [59]. Rigid bonds are non-flexible bonds that confer conformational stability. While such rigidity helps preserve the binding-competent conformation, excessive structural rigidity may diminish solubility and hinder membrane permeation [60]. All these parameters determine the fundamental pharmacokinetic and pharmacodynamic properties of a drug candidate molecule.

In addition to the direct anti-inflammatory activity of the compounds, the potential pathways contributing to this activity have also been investigated. Given the compounds’ lower probability of inhibiting proinflammatory cytokines such as TNF-α and ILs in our in silico studies, the observed anti-inflammatory activities are likely mediated by alternative and distinct mechanisms. The compounds demonstrated potential for immunomodulatory and immunostimulatory activities, enhanced TP53 expression, and acted as agonists for membrane integrity while inhibiting membrane permeability. Our in vitro findings demonstrate the stabilization of the red blood cell membrane, which strongly corroborates the predicted effects of membrane integrity agonists and permeability inhibitors identified through in silico analyses. The structural and functional integrity of the red blood cell membrane is closely correlated with the progression of inflammation-related atherosclerosis. Therefore, cardioprotective and hemostatic effects of the compounds were also analyzed. Most of the compounds we investigated exhibited positive effects on these endpoints. Particularly, hyperoside (**1**), isoquercetin (**2**), cichoriin (**8**)**,** isoorientin (**10**), and swertisin (**11**) exhibited a higher probability of enhancing TP53 expression, acting as membrane integrity agonists, inhibiting membrane permeability, providing cardioprotective and hemostatic effects. Overall, our in silico results align with and support the observations from the in vitro assays.

## 4. Materials and Methods

### 4.1. Plant Material

**Figure 4:**
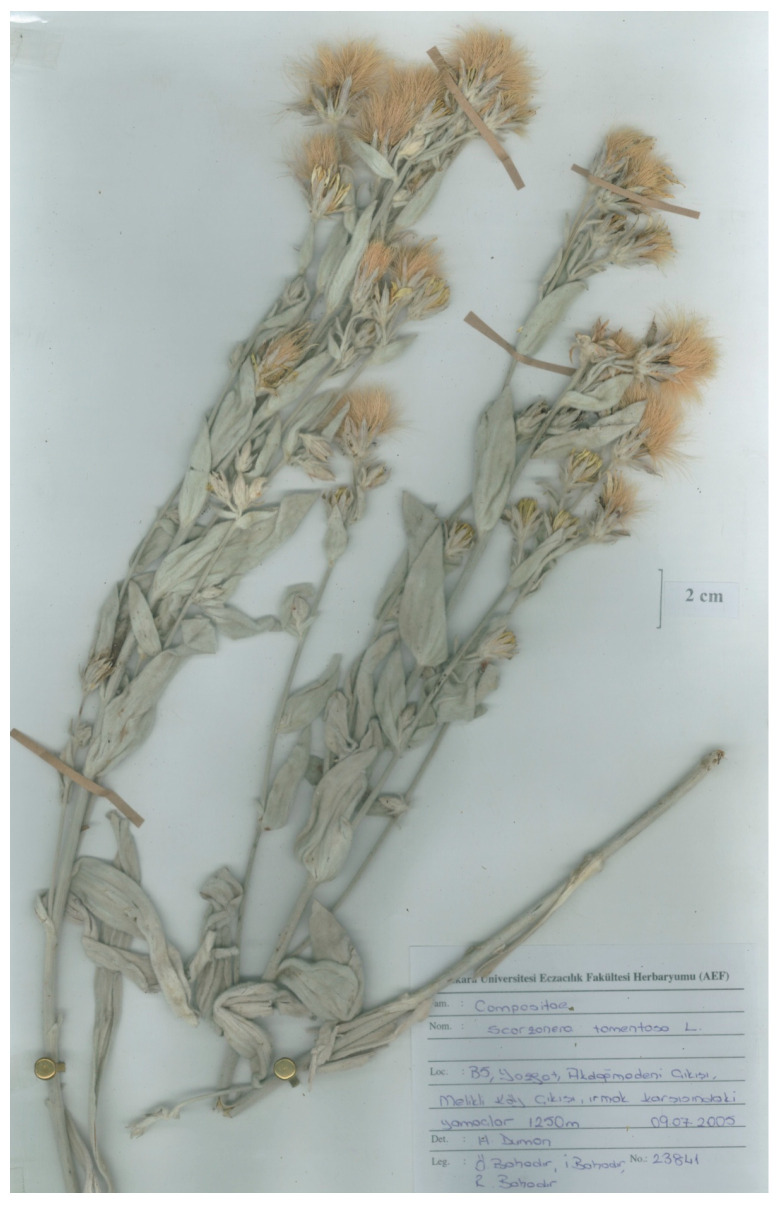
Herbarium sample of *S. tomentosa*.

*G. tomentosa* was collected from Yozgat-Akdağmadeni in July 2013, (N 40°28′13.253″ E 32°39′0.73″). Identification of the plant was carried out by Prof. Hayri Duman from Gazi University, Faculty of Science, Department of Biology as *Scorzonera tomentosa* L. The voucher specimen (Figure 4) was placed in the Ankara University Faculty of Pharmacy Herbarium (AEF 23841).

### 4.2. Extraction

Aerial parts were used for extraction. After drying and pulverizing the *G. tomentosa* aerial parts (750 g), the methanolic extract was prepared by maceration at room temperature for 24 h, followed by extraction in an ultrasonic bath for one hour. After filtration, the methanolic extract was completely dried under vacuum to obtain a crude extract through evaporation. Fractionation by liquid–liquid extraction was applied. The crude methanolic extract (131.0 g) was suspended in water (500 mL) and extracted with *n*-hexane, dichloromethane, and ethylacetate, respectively, to obtain four different phases as *n*-hexane (61.0 g), dichloromethane (15.1 g), ethylacetate (18.2 g), and the remaining water part of the methanolic extract (43.2 g). The ethyl acetate phase was selected for further studies due to its highest determined anti-inflammatory activity.

### 4.3. Isolation

Initially, the ethyl acetate phase (18.2 g) was separated by open column chromatography on silica gel 60 (70–230 mesh, 0.063–0.200 mm). Isolation was performed by elution with an ethylacetate/methanol/water (100:13.5:10) solvent system. The ethylacetate phase was separated into fractions, and they were investigated for their phytochemical profiles by TLC on silica gel 60 F254 (Merck, Darmstadt, Germany, 20 cm × 20 cm) precoated plates using an ethylacetate/methanol/water (100:13.5:10) solvent system. The plates were then examined under UV light, and Vanillin-H_2_SO_4_ reagent was used for detection. Fractions were combined according to their phytochemical profiles. Fraction STAE4, which was determined as the highest active fraction obtained from column chromatography, was subjected to separation by semi-prep HPLC on an ACE 5 C18 HPLC column eluting with a mobile phase made up of an ACN and water mixture in a gradient system to yield responsible compounds.

On the other hand, the dichloromethane phase, which was also found to have significant anti-inflammatory activity, was applied to column chromatography on silica gel 60 (70–230 mesh, 0.063–0.200 mm). A methanol/dichloromethane mixture of increasing polarity was used for elution. Fractions were analyzed in a similar way to determine their phytochemical contents using TLC on silica gel 60 F254 (20 × 20) precoated plates using a methanol/dichloromethane (20:80) solvent system.

### 4.4. In Vitro Analysis of the Putative Anti-Inflammatory Activity

The human red blood cell membrane-stabilizing activity of the plant extracts and isolated compounds against heat-induced hemolysis was investigated as an indicator of anti-inflammatory potential [61,62]. The study protocol was approved by the ethics committees of the Faculty of Medicine of Ankara University (14.05.2020/I5-273-20). For this purpose, informed consent and approval were obtained from the participants first. The procedures employed adhere to the Declaration of Helsinki. Fresh whole human blood was collected from healthy subjects who had not taken any steroid, analgesic, or anti-inflammatory medications for at least two weeks prior to the experiment. Centrifugation was performed to separate whole blood into its components at 3000 rpm for 10 min. Then, the supernatant was removed. The cell pellet was washed with sterile saline solution (0.85% *w*/*v* NaCl, pH 7.2) at least three times. The packed cell volume was measured, and the cells were reconstituted to a 10% *v*/*v* suspension in an isotonic buffer solution (pH 7.2). The reaction mixture consisted of different concentrations of test samples and 10% cell suspension. Then, incubation was performed at 56 °C for 30 min. At the end of the incubation, the tubes were cooled, and the reaction mixture was centrifuged at 2500 rpm for 5 min. The absorbance was measured at 560 nm. The activity of the solvent used to dissolve the extract or pure compound was also evaluated and designated as the solvent control. ASA was used as the positive control. The experiment was performed in triplicate. The percentages of hemolysis and protection were calculated using the following formula: percentage of hemolysis is “(Absorbance of test sample/Absorbance of control) × 100”, percentage of protection is “100 − [(Absorbance of test sample/Absorbance of control) × 100”. The half-maximal inhibitory concentration (IC_50_) was calculated from the percentage protection values using a calibration curve.

### 4.5. Statistical Analysis

Statistical data analysis was carried out using IBM SPSS Statistics version 25.0 (IBM Corp., Armonk, NY, USA). Results were expressed as mean ± standard deviation (SD). The Shapiro–Wilks test was used to assess the distribution of the data. Statistical comparisons were performed using a one-way analysis of variance (ANOVA) followed by the post hoc Tukey’s test. A *p*-value less than 0.05 was considered statistically significant.

### 4.6. In Silico Analysis

Canonical and isomeric SMILES, molecular weight, molecular formula, and structure of the isolated compounds were retrieved from online databases PubChem [63] and ChEMBL [64].

#### 4.6.1. Physicochemical and ADME/Pharmacokinetic Predictions

ADME profiles of the compounds were evaluated using ADMETlab 3.0-2025 [36,37]. Isomeric SMILES of each compound were employed for this evaluation. Structural and physicochemical properties, including MW, density, volume, solubility (logS, logP, logD 7.4, pKa acid and base), melting and boiling points, and molecular descriptors such as the number of Hacc, Hdon, rotatable bonds, and ring characteristics, were analyzed. Medicinal chemistry profiles, including QED, Lipinski’s Rule of Five, Pfizer’s Rule, GSK’s Rule, and the Golden Triangle Rule, were investigated. ADMETLab 3.0 has explained these rules as follows: Lipinski’s Rule MW ≤ 500, logP ≤ 5, Hacc ≤ 10, Hdon ≤ 5; Pfizer’s Rule logP > 3, TPSA < 75; GSK’s Rule MW ≤ 400, logP ≤ 4; the Golden Triangle Rule 200 ≤ MW ≤ 500, −2 ≤ logD ≤ 5. Caco-2, MDCK, and PAMPA permeability, P-gp inhibitor or substrate status, human intestinal absorption rate, and oral bioavailability (20%, 30%, and 50%) were evaluated to assess absorption. PPB, BBB, VDss, Fu, inhibitor effects on MRP1, OATP1B1, and OATP1B3 were investigated to determine distribution. The inhibitory or substrate impacts on various CYPs, including CYP3A4, 1A2, 2C9, 2C19, 2D6, 2B6, 2C8, and their human liver microsomal stability were investigated to evaluate metabolism. CLplasma and T_1/2_ were performed to determine excretion.

#### 4.6.2. Anti-Inflammatory Activity, Mechanism, and Target Prediction

The prediction of activity spectra for substances (PASS) software (v.2.0, 2024) was employed to assess the anti-inflammatory potential of the compounds by identifying inflammation-associated mechanisms and pathways [65,66]. Firstly, anti-inflammatory, immunomodulatory, immunostimulatory, TP53 expression-enhancing, and TNF expression-inhibitory potential was investigated. The antagonistic effects of the compounds on several ILs, including IL-1, IL-1α, IL-1*β*, IL-4, IL-5, IL-6, and IL-10, were examined. Then, membrane integrity, membrane permeability, cardioprotective, and hemostatic effects were predicted. The probability of being active (Pa) and the probability of being inactive (Pi) values were used to estimate the activity potential of the compounds. The results are shown as Pa and Pi values.

## 5. Conclusions

This study elucidates the anti-inflammatory properties of *G. tomentosa* aerial parts, focusing on the bioactive compounds isolated via bioactivity-guided fractionation through both in vitro and in silico methodologies. Analysis revealed a prominent presence of flavonoid glycosides and dicaffeoylquinic acid derivatives in the plant material. Among the isolated compounds, 4,5-*O*-dicaffeoylquinic acid demonstrated the highest activity in vitro test model; however, this was still inferior to that of the reference compound and the bioactive fraction STAE4. The isolated compounds exhibited enhanced anti-inflammatory effects when they were present together as in fraction STAE4, surpassing even that of the reference compound. The findings suggest that both flavonoids and dicaffeoylquinic acid derivatives contribute variably to the anti-inflammatory activity observed and imply a potential cooperative mechanism of action among the components in mitigating inflammation.

In conclusion, the flavonoid derivatives and dicaffeoylquinic acid derivatives identified in the active fraction collectively play significant roles by combating inflammation in *G. tomentosa* aerial parts. The anti-inflammatory efficacy of the compounds could be achieved primarily by protective effects on cell membrane rather than by inhibiting inflammatory mediators. In-depth investigations into their mechanisms of action will yield comprehensive insights into the pathways through which they mediate the inhibition of inflammatory processes.

## Figures and Tables

**Table 1:** Potential anti-inflammatory activities of -plant extracts.

Plant Name	IC_50_ (mg/mL)
*Podospermum canum*	5.81 ± 0.01 *
*Gelasia latifolia*	5.85 ± 0.04 *
*Pseudopodospermum szowitzii*	6.00 ± 0.08 *
*Gelasia cinerea*	6.72 ± 0.05 *
*Scorzonera parviflora*	6.06 ± 0.02 *
*Gelasia tomentosa*	5.71 ± 0.05 *
Solvent control	-
Positive control (ASA)	0.30 ± 0.01 *

Data are presented as mean ± SD from three replicates * *p* < 0.05; significantly different from the solvent control (one-way ANOVA followed by Tukey’s post hoc test).

**Table 2:** Potential anti-inflammatory activities of the phases of *Gelasia tomentosa* aerial parts.

Extract Name	IC_50_ (mg/mL)
Ethyl acetate phase	0.98 ± 0.01 *
Chloroform phase	0.98 ± 0.03 *
*n*-Hexane phase	2.94 ± 0.34 *
Remains water phase	3.05 ± 0.16 *
Solvent control	-
Positive control (ASA)	0.32 ± 0.00 *

Data are presented as mean ± SD from three replicates * *p* < 0.05; significantly different from the solvent control (one-way ANOVA followed by Tukey’s post hoc test).

**Table 3:** Potential anti-inflammatory activities of fractions obtained from the chloroform phase of *Gelasia tomentosa*.

Fractions	IC_50_ (mg/mL)
STC1	0.56 ± 0.00 *
STC2	0.82 ± 0.01 *
STC3	0.41 ± 0.00 *
STC4	0.40 ± 0.00 *
STC5	0.45 ± 0.00 *
STC6	0.56 ± 0.01 *
STC7	0.67 ± 0.01 *
STC8	0.55 ± 0.01 *
STC9	0.53 ± 0.01 *
Solvent control	-
Positive control (ASA)	0.31 ± 0.00 *

Data are presented as mean ± SD from three replicates * *p* < 0.05; significantly different from the solvent control (one-way ANOVA followed by Tukey’s post hoc test).

**Table 4:** Potential anti-inflammatory activities of fractions obtained from the ethyl acetate phase of *Gelasia tomentosa*.

Fractions	IC_50_ (mg/mL)
STAE1	0.41 ± 0.04 *
STAE2	0.37 ± 0.03 *
STAE3	0.45 ± 0.02 *
STAE4	0.16 ± 0.00 *
STAE5	0.28 ± 0.03 *
STAE6	0.37 ± 0.04 *
STAE7	0.31 ± 0.00 *
STAE8	0.26 ± 0.02 *
STAE9	0.21 ± 0.00 *
Solvent control	-
Positive control (ASA)	0.31 ± 0.00 *

Data are presented as mean ± SD from three replicates * *p* < 0.05; significantly different from the solvent control (one-way ANOVA followed by Tukey’s post hoc test).

**Table 5:** Putative anti-inflammatory activities of the isolated compounds.

Compounds	IC_50_ (mg/mL)
Hyperoside	0.87 ± 0.00 *
Isoquercetin	>1 mg/mL
Quercetin-3-*O*-*β*-apiofuranosyl-(1→2)-*β*-galactopyranoside	0.92 ± 0.03 *
Quercetin-3-*O*-*β*-apiofuranosyl-(1→2)-*β*-glucopyranoside	>1 mg/mL
7-Methoxyapigenin-6-*C*-*β*-apiofuranosyl-(1→2)-*β*-glucopyranoside	0.83 ± 0.00 *
Apigenin-6-*C*-*β*-apiofuranosyl-(1→2)-*β*-glucopyranoside	>1 mg/mL
Dihydrodehydrodiconiferyl alcohol-4-*O*-*β*-glucopyranoside	>1 mg/mL
Cichoriin	>1 mg/mL
7-*O*-Methylisoorientin	0.87 ± 0.00 *
Isoorientin	0.95 ± 0.00 *
Swertisin	>1 mg/mL
3,5-*O*-Dicaffeoylquinic acid methyl ester	0.83 ± 0.01 *
4,5-*O*-Dicaffeoylquinic acid methyl ester	0.92 ± 0.02 *
Staphylinioside E	>1 mg/mL
3,5-*O*-Dicaffeoylquinic acid	0.87 ± 0.01 *
4,5-*O*-Dicaffeoylquinic acid	0.55 ± 0.00 *
Solvent control	-
Positive control (ASA)	0.29 ± 0.01 *

Data are presented as mean ± SD from three replicates * *p* < 0.05; significantly different from the solvent control (one-way ANOVA followed by Tukey’s post hoc test).

**Table 6:** Anti-inflammatory and putative inflammation-related activity predictions of the isolated compounds.

Compounds	Anti-Inflammatory		Immunomodulator		Immunostimulant		TP53 Expression Enhancer		TNF Expression Inhibitor	
	Pa	Pi	Pa	Pi	Pa	Pi	Pa	Pi	Pa	Pi
**1**	0.739	0.011	0.325	0.057	0.594	0.024	0.959	0.003	N/A	N/A
**2**	0.739	0.011	0.325	0.057	0.594	0.024	0.959	0.003	N/A	N/A
**3**	0.770	0.009	N/A	N/A	0.734	0.012	0.888	0.006	N/A	N/A
**4**	0.770	0.009	N/A	N/A	0.734	0.012	0.888	0.006	N/A	N/A
**5**	0.549	0.044	N/A	N/A	0.222	0.170	0.871	0.006	N/A	N/A
**6**	0.572	0.038	N/A	N/A	0.610	0.022	0.886	0.006	N/A	N/A
**7**	0.752	0.010	N/A	N/A	0.514	0.035	0.614	0.045	N/A	N/A
**8**	0.733	0.012	0.386	0.032	0.512	0.035	0.873	0.006	N/A	N/A
**9**	0.321	0.142	N/A	N/A	0.210	0.124	0.807	0.010	0.231	0.160
**10**	0.496	0.058	N/A	N/A	0.397	0.054	0.962	0.003	0.335	0.082
**11**	0.443	0.076	N/A	N/A	0.343	0.065	0.956	0.003	0.371	0.067
**12**	0.702	0.016	N/A	N/A	0.170	0.154	0.750	0.017	0.534	0.023
**13**	0.703	0.015	N/A	N/A	N/A	N/A	0.719	0.022	0.567	0.017
**14**	0.447	0.074	0.283	0.080	0.639	0.019	0.786	0.012	0.315	0.093
**15**	0.657	0.022	N/A	N/A	0.220	0.118	0.705	0.025	0.552	0.019
**16**	0.660	0.021	N/A	N/A	0.209	0.125	0.665	0.033	0.585	0.015

N/A: not available; Pa: probability to be active; Pi: probability to be inactive; TNF: tumor necrosis factor; TP53: tumor protein.

**Table 7:** Anti-inflammatory and inflammation-related activity predictions of the isolated compounds.

Compounds	IL Antagonist		IL-1 Antagonist		IL-1α Antagonist		IL-1*β* Antagonist		IL-4 Antagonist		IL-5 Antagonist		IL-6 Antagonist		IL-10 Antagonist	
	Pa	Pi	Pa	Pi	Pa	Pi	Pa	Pi	Pa	Pi	Pa	Pi	Pa	Pi	Pa	Pi
**1**	N/A	N/A	N/A	N/A	N/A	N/A	N/A	N/A	0.423	0.005	N/A	N/A	0.341	0.010	0.104	0.061
**2**	N/A	N/A	N/A	N/A	N/A	N/A	N/A	N/A	0.423	0.005	N/A	N/A	0.341	0.010	0.104	0.061
**3**	N/A	N/A	N/A	N/A	N/A	N/A	N/A	N/A	0.284	0.010	N/A	N/A	0.248	0.040	N/A	N/A
**4**	N/A	N/A	N/A	N/A	N/A	N/A	N/A	N/A	0.284	0.010	N/A	N/A	0.248	0.040	N/A	N/A
**5**	N/A	N/A	N/A	N/A	N/A	N/A	N/A	N/A	0.247	0.013	N/A	N/A	N/A	N/A	N/A	N/A
**6**	N/A	N/A	N/A	N/A	N/A	N/A	N/A	N/A	0.285	0.010	N/A	N/A	N/A	N/A	N/A	N/A
**7**	N/A	N/A	N/A	N/A	N/A	N/A	N/A	N/A	N/A	N/A	N/A	N/A	N/A	N/A	N/A	N/A
**8**	N/A	N/A	N/A	N/A	N/A	N/A	N/A	N/A	N/A	N/A	N/A	N/A	0.453	0.005	N/A	N/A
**9**	N/A	N/A	N/A	N/A	N/A	N/A	N/A	N/A	0.157	0.042	N/A	N/A	N/A	N/A	0.100	0.068
**10**	N/A	N/A	N/A	N/A	N/A	N/A	N/A	N/A	0.429	0.005	N/A	N/A	N/A	N/A	N/A	N/A
**11**	N/A	N/A	N/A	N/A	N/A	N/A	N/A	N/A	0.387	0.005	N/A	N/A	N/A	N/A	0.100	0.069
**12**	N/A	N/A	N/A	N/A	N/A	N/A	N/A	N/A	0.152	0.047	N/A	N/A	0.193	0.087	N/A	N/A
**13**	0.216	0.069	0.134	0.076	0.088	0.049	0.074	0.070	0.176	0.030	0.111	0.031	0.254	0.036	0.124	0.032
**14**	N/A	N/A	N/A	N/A	0.082	0.066	N/A	N/A	N/A	N/A	N/A	N/A	N/A	N/A	N/A	N/A
**15**	0.144	0.135	N/A	N/A	0.082	0.066	N/A	N/A	0.152	0.047	N/A	N/A	0.193	0.087	N/A	N/A
**16**	0.151	0.125	N/A	N/A	0.000	0.000	N/A	N/A	0.133	0.070	0.085	0.074	0.216	0.065	0.120	0.036

IL: interleukin; N/A: not available; Pa: probability to be active; Pi: probability to be inactive.

**Table 8:** Membrane integrity, membrane permeability, cardioprotective and hemostatic effects of the isolated compounds.

Compounds	MIA		MPI		Cardioprotectant		Hemostatic	
	Pa	Pi	Pa	Pi	Pa	Pi	Pa	Pi
**1**	0.989	0.001	0.981	0.001	0.984	0.001	0.987	0.001
**2**	0.989	0.001	0.981	0.001	0.984	0.001	0.987	0.001
**3**	0.970	0.002	0.949	0.002	0.909	0.003	0.740	0.003
**4**	0.970	0.002	0.949	0.002	0.909	0.003	0.740	0.003
**5**	0.909	0.009	0.707	0.036	0.789	0.004	0.365	0.014
**6**	0.922	0.006	0.762	0.018	0.811	0.003	0.422	0.009
**7**	0.895	0.012	0.680	0.048	0.552	0.008	0.405	0.010
**8**	0.955	0.003	0.925	0.003	0.849	0.003	0.950	0.002
**9**	0.907	0.009	0.647	0.063	0.667	0.004	0.338	0.018
**10**	0.957	0.003	0.893	0.004	0.963	0.002	0.676	0.003
**11**	0.957	0.003	0.831	0.006	0.957	0.002	0.593	0.004
**12**	0.955	0.003	0.878	0.004	0.276	0.104	0.254	0.049
**13**	0.935	0.004	0.856	0.005	N/A	N/A	0.202	0.097
**14**	0.825	0.031	0.734	0.026	0.375	0.035	0.331	0.020
**15**	0.947	0.004	0.844	0.005	0.276	0.103	0.278	0.036
**16**	0.924	0.006	0.821	0.007	N/A	N/A	0.223	0.074

MIA: membrane integrity agonist; MPI: membrane permeability inhibitor; N/A: not available; Pa: probability to be active; Pi: probability to be inactive.

## Data Availability

The original contributions presented in this study are included in the article and Supplementary Material. Further inquiries can be directed to the corresponding author.

## Supplementary Materials

The following supporting information can be downloaded at https://www.mdpi.com/article/10.3390/molecules31010019/s1, Table S1: Names, SMILES, and molecular information about the isolated compounds; Table S2: Predicted physicochemical properties of the compounds—1; Table S3: Predicted physicochemical properties of the compounds—2; Table S4: Predicted medicinal chemistry properties of the compounds; Table S5: Predicted absorption and distribution properties of the compounds; Table S6: Predicted metabolism and excretion properties of the compounds.

## References

[1] Rote N.S., Huether S.E., McCance K.L., Huether S.E., Brashers V.L., Rote N.S. (2010). Innate immunity: Inflammation. Pathophysiology: The Biological Basis for Disease in Adults and Children.

[2] Hall J.E. (2011). Guyton and Hall Textbook of Medical Physiology.

[3] Ashley N.T., Weil Z.M., Nelson R.J. (2012). Inflammation: Mechanisms, costs, and natural variation. Annu. Rev. Ecol. Evol. Syst..

[4] Ereminsoy E. (2014). *Maclura pomifera* Plant Obtained from the Water Extract Carrageenan-Induced Inflammation in Rats Model and Investigation of the Effect on Cytokines. Master’s Thesis.

[5] Aggarwal B.B., Shishodia S., Sandur S.K., Pandey M.K., Sethi G. (2006). Inflammation and cancer: How hot is the link?. Biochem. Pharmacol..

[6] Sung B., Prasad S., Gupta S.C., Patchva S., Aggarwal B.B., Shyur L.F., Lau S.Y.A. (2012). Regulation of inflammation-mediated chronic diseases by botanicals. Advances in Botanical Research.

[7] Rayburn E.R., Ezell S.J., Zhang R. (2009). Anti-inflammatory agents for cancer therapy. Mol. Cell Pharmacol..

[8] Lmadani Y.H., Vorstenbosch J., Davison P.G., Murphy A.M. (2021). Wound healing: A comprehensive review. Semin. Plast. Surg..

[9] Shukla S.K., Sharma A.K., Gupta V., Yashavarddhan M.H. (2019). Pharmacological control of inflammation in wound healing. J. Tissue Viability.

[10] Chamberlain D.F., Davis P.H. (1975). *Scorzonera* L.. Flora of Turkey and the East Aegean Islands.

[11] Şenkardeş İ., Bulut G., Doğan A., Tuzlacı E. (2019). An ethnobotanical analysis on wild edible plants of the Turkish Asteraceae Taxa. Agric. Conspec. Sci..

[12] Baytop T. (1999). Therapy with Medicinal Plants in Turkey: Past and Present.

[13] Sezik E., Yeşİlada E., Tabata M., Honda G., Takaishi Y., Fujita T., Tanaka T., Takeda Y. (1997). Traditional medicine in Turkey VIII. Folk medicine in East Anatolia; Erzurum, Erzíncan, Agrı, Kars, Igdir Provinces. Econ. Bot..

[14] Altundağ E., Öztürk M. (2011). Ethnobotanical studies on the plant resources of East Anatolia, Turkey, Procedia. Soc. Behav. Sci..

[15] Karakaya S., Polat A., Aksakal Ö., Sümbüllü Y.Z., İncekara Ü. (2020). Ethnobotanical study of medicinal plants in Aziziye district (Erzurum, Turkey). Turk. J. Pharm. Sci..

[16] Demirci S., Özhatay N. (2012). An ethnobotanical study in Kahramanmaraş (Turkey); wild plants used for medicinal purpose in Andırın, Kahramanmaraş. Turk. J. Pharm. Sci..

[17] Akkol E.K., Acıkara O.B., Süntar I., Citoğlu G.S., Keleş H., Ergene B. (2011). Enhancement of wound healing by topical application of *Scorzonera* species: Determination of the constituents by HPLC with new validated reverse phase method. J. Ethnopharmacol..

[18] Bahadır Ö., Saltan H.G., Özbek H. (2012). Antinociceptive activity of some *Scorzonera* L. species. Turk. J. Med. Sci..

[19] Özbek H., Bahadır Acıkara Ö., Keskin İ., Kırmızı N.İ., Yiğitbaşı T., Sakul A.A., İşcan G.S. (2017). Preclinical Evaluation of *Scorzonera* sp. root extracts and major compounds against acute hepatotoxicity ınduced by carbon tetrachloride. Indian J. Pharm. Sci..

[20] Bahadır Acikara Ö., Hošek J., Babula P., Cvačka J., Budešínský M., Dračinský M., Saltan İşcan G., Kadlecová D., Ballová L., Šmejkal K. (2016). Turkish *Scorzonera* species extracts attenuate cytokine secretion via inhibition of NF-kappaB activation, showing anti-inflammatory effects in vitro. Molecules.

[21] Dall’Acqua S., Ak G., Sut S., Ferrarese I., Zengin G., Yıldıztugay E., Mahomoodally M.F., Sinan K.I., Lobine D. (2020). Phenolics from *Scorzonera tomentosa* L.: Exploring the potential use in industrial applications via an integrated approach. Ind. Crop Prod..

[22] Karagöz A., Artun F.T., Özcan G., Melikoğlu G., Anıl S., Kültür Ş., Sütlüpınar N. (2015). In vitro evaluation of antioxidant activity of some plant methanol extracts. Biotechnol. Biotechnol. Equip..

[23] Bahadir-Acikara O., Küpeli-Akkol E., Süntar İ., Ergene B., Saltan-Çitoğlu G., Çoban T. (2014). Assessment of anti-ınflammatory and free radical scavenger activities of selected *Scorzonera* Species and determination of active components. Int. J. Pharmacogn. Phytochem. Res..

[24] Saracoğlu I., Harput Ü.Ş., Çaliş I., Ogihara Y. (2002). Phenolic Constituents from *Phlomis lycia*. Turk. J. Chem..

[25] Sukito A., Tachibana S. (2014). Isolation of hyperoside and isoquercitrin from *Camellia sasanqua* as antioxidant agents. Pak. J. Biol. Sci..

[26] Akkol E.K., Šmejkal K., Kurtul E., Ilhan M., Güragac F.T., İşcan G.S., Acıkara Ö.B., Cvačka J., Buděšínský M. (2019). Inhibitory activity of *Scorzonera latifolia* and its components on enzymes connected with the healing process. J. Ethnopharmacol..

[27] Vasilev H., Šmejkal K., Gronover C.S., Choi Y.H., Prüfer D., Jankovská D., Ionkova I. (2021). Flavonol glycosides from aerial parts of *Astragalus thracicus* Grıseb. Phytochem. Lett..

[28] Ma C., Nakamura N., Hattori M. (1998). Saponins and C-glycosyl flavones from the seeds of *Abrus precatorius*. Chem. Pharm. Bull..

[29] Feng W.S., Li K.K., Zheng X.K. (2011). A new norlignan lignanoside from *Selaginella moellendorffii* Hieron. Acta Pharm. Sinic.

[30] Matsuda N., Sato H., Yaoıta Y., Kıkuchi M. (1996). Isolation and absolute structures of the neolignan glycosides with the enantiometric aglycones from the leaves of *Viburnum awabuki* K. Koch. Chem. Pharm. Bull..

[31] Kisiel W., Michalska K. (2002). A new coumarin glucoside ester from *Cichorium intybus*. Fitoterapia.

[32] Tsevegsuren N., Proksch P., Wang Y., Davaakhuu G. (2011). Bioactive phenolic acids from *Scorzonera radiata* Fisch. Mong. J. Chem..

[33] Tabassum N., Lee J.H., Yim S.H., Batkhuu G.J., Jung D.W., Williams D.R. (2016). Isolation of 4,5-*O*-dicaffeoylquinic acid as a pigmentation inhibitor occurring in *Artemisia capillaris* thunberg and its validation in vivo. Evid. Based Complement. Altern. Med..

[34] Ge L., Wan H., Tang S., Chen H., Li J., Zhang K., Zhou B., Fei J., Wu S., Zeng X. (2018). Novel caffeoylquinic acid derivatives from *Lonicera japonica* Thunb. flower buds exert pronounced anti-HBV activities. RSC Adv..

[35] Yu Q., Matsunami K., Otsuka H., Takeda Y. (2005). Staphylionosides A-K:megastigmane glucosides from the leaves of *Staphylea bumalda* DC. Chem. Pharm. Bull..

[36] Dong J., Wang N.N., Yao Z.J., Zhang L., Cheng Y., Ouyang D., Lu A.P., Cao D.S. (2018). ADMETlab: A platform for systematic ADMET evaluation based on a comprehensively collected ADMET database. J. Cheminform.

[37] Fu L., Shi S., Yi J., Wang N., He Y., Wu Z., Peng J., Deng Y., Wang W., Wu C. (2024). ADMETlab 3.0: An updated comprehensive online ADMET prediction platform enhanced with broader coverage, improved performance, API functionality and decision support. Nucleic Acids Res..

[38] Lipinski C.A. (2004). Lead-and drug-like compounds: The rule-of-five revolution. Drug Discov. Today Technol..

[39] Li B., Wang Z., Liu Z., Tao Y., Sha C., He M., Li X. (2024). DrugMetric: Quantitative drug-likeness scoring based on chemical space distance. Brief. Bioinform..

[40] Sarı A., Zidorn C., Ellmerer E.P., Özgökçe F., Ongania K.H., Stuppner H. (2007). Phenolic compounds from *Scorzonera tomentosa* L.. Helv. Chim. Acta.

[41] Erden Y., Kırbağ S., Yılmaz Ö. (2013). Phytochemical composition and antioxidant activity of some *Scorzonera* species. Proc. Natl. Acad. Sci. India Sect. B Biol. Sci..

[42] Bahadır-Acıkara Ö., Özbilgin S., Saltan-İşcan G., Dall’Acqua S., Rjašková V., Özgökçe F., Suchy V., Šmejkal K. (2018). Phytochemical analysis of *Podospermum* and *Scorzonera n*-hexane extracts and the HPLC quantitation of triterpenes. Molecules.

[43] Bahadır Acıkara Ö., Saltan Çitoğlu G., Gençler Özkan A.M. (2013). Qualitative and quantitative analysis of phenolic acids in *Scorzonera tometosa* L.. Turk. J. Pharm. Sci..

[44] Granica S., Lohwasser U., Jöhrer K., Zidorn C. (2015). Qualitative and quantitative analyses of secondary metabolites in aerial and subaerial of *Scorzonera hispanica* L. (black salsify). Food Chem..

[45] Sareedenchai V., Zidorn C. (2010). Flavonoids as chemo systematic markers in the tribe Cichorieae of the Asteraceae. Biochem. Syst. Ecol..

[46] Maleki S.J., Crespo J.F., Cabanillas B. (2019). Antiinflammatory effects of flavonoids. Food Chem..

[47] Abdel Motaal A., Ezzat S.M., Tadros M.G., El-Askary H.I. (2016). In vivo anti-inflammatory activity of caffeoylquinic acid derivatives from *Solidago virgaureat* in rats. Pharm. Biol..

[48] Wan P., Xie M., Chen G., Dai Z., Hu B., Zeng X., Sun Y. (2019). Anti-inflammatory effects of dicaffeoylquinic acids from *Ilex kudingcha* on lipopolysaccharide-treated RAW264.7 macrophages and potential mechanisms. Food Chem. Toxicol..

[49] Song J., Li P., Yu Y., Kim J., Chun H. (2023). Antioxidant and anti-inflammatory effects of 6,3′,4′- and 7,3′,4′-trihydroxyflavone on 2D and 3D RAW264.7 models. Antioxidants.

[50] Li J., Zhao R., Miao P., Xu F., Chen J., Jiang X., Hui Z., Wang L., Bai R. (2023). Discovery of anti-inflammatory natural flavonoids: Diverse scaffolds and promising leads for drug discovery. Eur. J. Med. Chem..

[51] Zheleva-Dimitrova D., Simeonova R., Kondeva-Burdina M., Savov Y., Balabanova V., Zengin G., Petrova A., Gevrenova R. (2023). Antioxidant and Hepatoprotective Potential of *Echinops ritro* L. Extracts on Induced Oxidative Stress In Vitro/In Vivo. Int. J. Mol. Sci..

[52] Aidoo D.B., Konja D., Henneh I.T., Ekor M. (2021). Protective Effect of Bergapten against Human Erythrocyte Hemolysis and Protein Denaturation In Vitro. Int. J. Inflamm..

[53] Anosike C.A., Obidoa O., Ezeanyika L.U.S. (2012). Membrane stabilization as a mechanism of the anti-inflammatory activity of some plants. DARU J. Pharm. Sci..

[54] Yesmin F., Paul A., Naz T., Atiqur Rahman A.B.M., Akhter S.F., Wahed M.I.I., Emran T.B., Siddiqui S.A. (2020). Membrane stabilization as a mechanism of anti-inflammatory action: A review. Clin. Phytosci..

[55] Kenny P.W. (2022). Hydrogen-bond donors in drug design. J. Med. Chem..

[56] Coimbra J.T., Feghali R., Ribeiro R.P., Ramos M.J., Fernandes P.A. (2021). The importance of intramolecular hydrogen bonds on the translocation of the small drug piracetam through a lipid bilayer. RSC Adv..

[57] Caron G., Digiesi V., Solaro S., Ermondi G. (2020). Flexibility in early drug discovery: Focus on the beyond-Rule-of-5 chemical space. Drug Discov. Today.

[58] Asano D., Takakusa H., Nakai D. (2023). Oral absorption of middle-to-large molecules and its improvement, with a focus on new modality drugs. Pharmaceutics.

[59] Ritchie T.J., Macdonald S.J. (2009). The impact of aromatic ring count on compound developability–are too many aromatic rings a liability in drug design?. Drug Discov. Today.

[60] Lawson A.D., MacCoss M., Heer J.P. (2017). Importance of rigidity in designing small molecule drugs to tackle protein–protein interactions (PPIs) through stabilization of desired conformers: Miniperspective. J. Med. Chem..

[61] Gunathilake K.D.P.P., Ranaweera K.K.D.S., Rupasinghe H.P. (2018). In vitro anti-inflammatory properties of selected green leafy vegetables. Biomedicine.

[62] Shinde U.A., Phadke A.S., Nair A.M., Mungantiwar A.A., Dikshit V.J., Saraf M.N. (1999). Membrane stabilizing activity—A possible mechanism of action for the anti-inflammatory activity of *Cedrus deodara* wood oil. Fitoterapia.

[63] PubChem Database; National Center for Biotechnology Information. https://pubchem.ncbi.nlm.nih.gov.

[64] ChEMBL Database; European Bioinformatics Institute (EMBL-EBI). https://www.ebi.ac.uk/chembl/.

[65] Filimonov D.A., Lagunin A.A., Gloriozova T.A., Rudik A.V., Druzhilovskii D.S., Pogodin P.V., Poroikov V.V. (2014). Prediction of the biological activity spectra of organic compounds using the PASS online web resource. Chem. Heterocycl. Compd..

[66] Poroikov V.V., Filimonov D.A., Gloriozova T.A., Lagunin A.A., Druzhilovskiy D.S., Rudik A.V., Stolbov L.A., Dmitriev A.V., Tarasova O.A., Ivanov S.M. (2019). Computer-aided prediction of biological activity spectra for organic compounds: The possibilities and limitations. Russ. Chem. Bull..

