# Supplementary material for: Discovering the Anti-Inflammatory Potential of Compounds Isolated from the Aerial Parts of Gelasia tomentosa (L.) Zaika, Sukhor. & N.Kilian (Syn. Scorzonera tomentosa), Through In Vitro Techniques and Advanced In Silico Modeling Approaches
Source: Molecules. 2025 Dec 20;31(1):19. doi: 10.3390/molecules31010019 (PMC12786445; doi:10.3390/molecules31010019)
Supplement: Supplementary file 1 [file molecules-31-00019-s001.zip › molecules-4007622-supplementary.pdf]

Table S1: Names, SMILES, and molecular information about the isolated compounds

No	Compounds	Canonical SMILES / Isomeric SMILES	MF	MW g/mol
**1**	Hyperoside	C1=CC(=C(C=C1C2=C(C(=O)C3=C(C=C(C=C3O2)O)O)OC4C(C(C(C(O4)CO)O)O)O)O)O<br>/<br>C1=CC(=C(C=C1C2=C(C(=O)C3=C(C=C(C=C3O2)O)O)O[C@H]4[C@@H]([C@H]([C@H]([C@H](O4)CO)O)O)O)O)O	$C_{21}H_{20}O_{12}$	464.4
**2**	Isoquercetin	C1=CC(=C(C=C1C2=C(C(=O)C3=C(C=C(C=C3O2)O)O)OC4C(C(C(C(O4)CO)O)O)O)O)O<br>/<br>C1=CC(=C(C=C1C2=C(C(=O)C3=C(C=C(C=C3O2)O)O)O[C@H]4[C@@H]([C@H]([C@@H]([C@H](O4)CO)O)O)O)O)O	$C_{21}H_{20}O_{12}$	464.4
**3**	Quercetin-3-*O*-*β*-apiofuranosyl-(1→2)-*β*-galactopyranoside	O=C1C(OC2OC(CO)C(O)C(O)C2OC3OCC(O)(CO)C3O)=C(OC=4C=C(O)C=C(O)C14)C=5C=CC(O)=C(O)C5<br>/<br>O(C1=C(OC=2C(C1=O)=C(O)C=C(O)C2)C3=CC(O)=C(O)C=C3)[C@H]4[C@H](O[C@H]5[C@H](O)[C@](CO)(O)CO5)[C@@H](O)[C@@H](O)[C@@H](CO)O4	$C_{26}H_{28}O_{16}$	596.5
**4**	Quercetin-3-*O*-*β*-apiofuranosyl-(1→2)-*β*-glucopyranoside	O=C1C(OC2OC(CO)C(O)C(O)C2OC3OCC(O)(CO)C3O)=C(OC=4C=C(O)C=C(O)C14)C=5C=CC(O)=C(O)C5<br>/<br>O(C1=C(OC=2C(C1=O)=C(O)C=C(O)C2)C3=CC(O)=C(O)C=C3)[C@H]4[C@H](O[C@H]5[C@H](O)[C@](CO)(O)CO5)[C@@H](O)[C@H](O)[C@@H](CO)O4	$C_{26}H_{28}O_{16}$	596.5
**5**	7-Methoxyapigenin-6-C-*β*-apiofuranosyl-(1→2)-*β*-glucopyranoside	O=C1C=C(OC=2C=C(OC)C(=C(O)C12)C3OC(CO)C(O)C(O)C3OC4OCC(O)(CO)C4O)C=5C=CC(O)=CC5<br>/<br>O([C@H]1[C@@H](O[C@H](CO)[C@@H](O)[C@@H]1O)C2=C(OC)C=C3C(=C2O)C(=O)C=C(O3)C4=CC=C(O)C=C4)[C@H]5[C@H](O)[C@](CO)(O)CO5	$C_{27}H_{30}O_{14}$	578.5

**6**	Apigenin-6-C-*β*-apiofuranosyl-(1→2)-*β*-glucopyranoside	O=C1C=C(OC2=CC(O)=C(C(O)=C12)C3OC(CO)C(O)C(O)C3OC4OCC(O)(CO)C4O)C=5C=CC(O)=CC5<br>/<br>OC=1C(=C(O)C=C2C1C(=O)C=C(O2)C3=CC=C(O)C=C3)[C@H]4[C@H](O[C@H]5[C@H](O)[C@](CO)(O)CO5)[C@@H](O)[C@H](O)[C@@H](CO)O4	$C_{26}H_{28}O_{14}$	564.5
**7**	Dihydrodehydrodiconiferyl alcohol-4-*O*-*β*-glucopyranoside	COC1=CC(=CC2=C1OC(C2CO)C3=CC(=C(C=C3)OC4C(C(C(C(O4)CO)O)O)O)OC)C=CCO<br>/<br>COC1=CC(=CC2=C1O[C@@H]([C@H]2CO)C3=CC(=C(C=C3)O[C@H]4[C@@H]([C@H]([C@@H]([C@H](O4)CO)O)O)O)OC)/C=C/CO	$C_{26}H_{32}O_{11}$	520.5
**8**	Cichoriin	C1=CC(=O)OC2=CC(=C(C=C21)O)OC3C(C(C(C(O3)CO)O)O)O<br>/<br>C1=CC(=O)OC2=CC(=C(C=C21)O)O[C@H]3[C@@H]([C@H]([C@@H]([C@H](O3)CO)O)O)O	$C_{15}H_{16}O_9$	340.3
**9**	7-*O*-Methylisoorientin	COC1=CC2C(C(=O)C=C(O2)C3=CC(=C(C=C3)O)O)C(=C1C4C(C(C(C(O4)CO)O)O)O)O<br>/<br>COC1=CC2C(C(=O)C=C(O2)C3=CC(=C(C=C3)O)O)C(=C1[C@H]4[C@@H]([C@H]([C@@H]([C@H](O4)CO)O)O)O)O	$C_{22}H_{24}O_{11}$	464.4
**10**	Isoorientin	C1=CC(=C(C=C1C2=CC(=O)C3=C(O2)C=C(C(=C3O)C4C(C(C(C(O4)CO)O)O)O)O)O)O<br>/<br>C1=CC(=C(C=C1C2=CC(=O)C3=C(O2)C=C(C(=C3O)[C@H]4[C@@H]([C@H]([C@@H]([C@H](O4)CO)O)O)O)O)O)O	$C_{21}H_{20}O_{11}$	448.4
**11**	Swertisin	COC1=C(C(=C2C(=C1)OC(=CC2=O)C3=CC=C(C=C3)O)O)C4C(C(C(C(O4)CO)O)O)O<br>/<br>COC1=C(C(=C2C(=C1)OC(=CC2=O)C3=CC=C(C=C3)O)O)[C@H]4[C@@H]([C@H]([C@@H]([C@H](O4)CO)O)O)O	$C_{22}H_{22}O_{10}$	446.4

**12**	3,5-*O*-Dicaffeoylquinic acid methyl ester	COC(=O)C1(CC(C(C(C1)OC(=O)C=CC2=CC(=C(C=C2)O)O)O)OC(=O)C=CC3=CC(=C(C=C3)O)O)O / COC(=O)C1(C[C@H](C([C@@H](C1)OC(=O)/C=C/C2=CC(=C(C=C2)O)O)O)OC(=O)/C=C/C3=CC(=C(C=C3)O)O)O	$C_{26}H_{26}O_{12}$	530.5
**13**	4,5-*O*-Dicaffeoylquinic acid methyl ester	COC(=O)C1(CC(C(C(C1)OC(=O)C=CC2=CC(=C(C=C2)O)O)OC(=O)C=CC3=CC(=C(C=C3)O)O)O)O / COC(=O)[C@]1(C[C@H]([C@@H]([C@@H](C1)OC(=O)/C=C/C2=CC(=C(C=C2)O)O)OC(=O)/C=C/C3=CC(=C(C=C3)O)O)O)O	$C_{26}H_{26}O_{12}$	530.5
**14**	Staphylinioside E	OCC1OC(OC2CC(C(C=CC(O)C)=C(C)C2O)(C)C)C(O)C(O)C1O / O([C@@H]1[C@H](O)C(C)=C(/C=C/[C@H](C)O)C(C)(C)C1)[C@@H]2O[C@H](CO)[C@@H](O)[C@H](O)[C@H]2O	$C_{19}H_{32}O_{8}$	388.5
**15**	3,5-*O*-Dicaffeoylquinic acid	C1C(C(C(CC1(C(=O)O)O)OC(=O)C=CC2=CC(=C(C=C2)O)O)O)OC(=O)C=CC3=CC(=C(C=C3)O)O / C1C(C[C@H](C([C@@H]1OC(=O)/C=C/C2=CC(=C(C=C2)O)O)O)OC(=O)/C=C/C3=CC(=C(C=C3)O)O)(O)C(=O)O	$C_{25}H_{24}O_{12}$	516.4
**16**	4,5-*O*-Dicaffeoylquinic acid	C1C(C(C(CC1(C(=O)O)O)OC(=O)C=CC2=CC(=C(C=C2)O)O)OC(=O)C=CC3=CC(=C(C=C3)O)O)O / C1[C@H]([C@H]([C@@H](C[C@@]1(C(=O)O)O)OC(=O)/C=C/C2=CC(=C(C=C2)O)O)OC(=O)/C=C/C3=CC(=C(C=C3)O)O)O	$C_{25}H_{24}O_{12}$	516.4

MF: molecular formula; MW: molecular weight

Table S2. Predicted physicochemical properties of the compounds - 1

Compounds	Volume	Density	nHA	nHD	nRot	nRing	MR	nHet	FC	nRig	Flex.	SCs	TPSA (Å²)
**1**	421.937	1.1	12.0	8.0	4.0	4.0	10.0	12.0	0.0	24.0	0.167	5.0	210.51
**2**	421.937	1.1	12.0	8.0	4.0	4.0	10.0	12.0	0.0	24.0	0.167	5.0	210.51
**3**	535.022	1.114	16.0	10.0	7.0	5.0	10.0	16.0	0.0	29.0	0.241	8.0	269.43
**4**	535.022	1.114	16.0	10.0	7.0	5.0	10.0	16.0	0.0	29.0	0.241	8.0	269.43
**5**	534.737	1.081	14.0	8.0	7.0	5.0	10.0	14.0	0.0	29.0	0.241	8.0	228.97
**6**	517.441	1.09	14.0	9.0	6.0	5.0	10.0	14.0	0.0	29.0	0.207	8.0	239.97
**7**	502.264	1.036	11.0	6.0	9.0	4.0	9.0	11.0	0.0	23.0	0.391	7.0	167.53
**8**	308.257	1.103	9.0	5.0	3.0	3.0	10.0	9.0	0.0	18.0	0.167	5.0	149.82
**9**	433.08	1.072	11.0	7.0	4.0	4.0	10.0	11.0	0.0	24.0	0.167	7.0	186.37
**10**	413.147	1.085	11.0	8.0	3.0	4.0	10.0	11.0	0.0	24.0	0.125	5.0	201.28
**11**	421.653	1.058	10.0	6.0	4.0	4.0	10.0	10.0	0.0	24.0	0.167	5.0	170.05
**12**	509.064	1.041	12.0	6.0	10.0	3.0	6.0	12.0	0.0	23.0	0.435	2.0	200.28
**13**	509.064	1.041	12.0	6.0	10.0	3.0	6.0	12.0	0.0	23.0	0.435	4.0	200.28
**14**	385.116	1.008	8.0	6.0	5.0	2.0	6.0	8.0	0.0	13.0	0.385	8.0	139.84
**15**	491.768	1.05	12.0	7.0	9.0	3.0	6.0	12.0	0.0	23.0	0.391	2.0	211.28
**16**	491.768	1.05	12.0	7.0	9.0	3.0	6.0	12.0	0.0	23.0	0.391	4.0	211.28

The names of the compounds indicated by numbers are as follows; hyperoside (1), isoquercetin (2), quercetin 3-*O*-*β*-apiofuranosyl-(1→2)-*β*-galactopyranoside (3), quercetin 3-*O*-*β*-apiofuranosyl-(1→2)-*β*-glucopyranoside (4), 7-methoxyapigenin-6-C-*β*-apiofuranosyl-(1→2)-*β*-glucopyranoside (5), apigenin-6-C-*β*-apiofuranosyl-(1→2)-*β*-glucopyranoside (6), dihydrodehydrodiconiferyl-alcohol-4-*O*-*β*-glucopyranoside (7), cichoriin (8), 7-*O*-methylisoorientin (9), isoorientin (10), swertisin (11), 3,5-*O*-dicaffeoylquinic acid methyl ester (12), 4,5-*O*-dicaffeoylquinic acid methyl ester (13), staphylinioside E (14), 3,5-*O*-dicaffeoylquinic acid (15), and 4,5-*O*-dicaffeoylquinic acid (16). The results determined to be optimal according to the model evaluation are marked in red. FC: formal charge; Flex: flexibility; HA: hydrogen bond acceptors; HD: hydrogen bond donors; Het: heteroatoms; MR: MaxRing; the number of atoms in the biggest ring; MW: molecular weight; n: number; Rot: rotatable bonds; SCs: stereo centers; TPSA: topological polar surface area.

Table S3. Predicted physicochemical properties of the compounds - 2

Compounds	logS (log mol/L)	logP (log mol/L)	logD7.4 (log mol/L)	pka (acid)	pka (base)	Melting point (°C)	Boiling point (°C)
**1**	-3.644	0.859	1.144	6.109	3.553	278.435	384.334
**2**	-3.63	0.697	1.087	5.983	3.818	270.754	381.207
**3**	-3.537	0.801	0.991	6.467	3.375	282.089	391.719
**4**	-3.598	0.637	0.837	6.573	3.599	287.848	398.743
**5**	-3.321	0.793	1.191	7.365	4.904	243.063	362.036
**6**	-3.384	0.635	0.784	6.331	4.592	256.043	381.036
**7**	-2.59	1.052	1.588	6.944	6.516	140.105	296.927
**8**	-1.746	-1.178	-0.115	6.905	3.535	216.192	370.915
**9**	-2.141	-0.555	0.099	7.213	5.293	206.243	341.122
**10**	-3.694	0.572	0.883	5.392	4.51	251.836	386.775
**11**	-3.377	1.07	1.475	6.719	5.278	216.336	329.898
**12**	-3.836	1.701	1.884	8.068	4.232	224.204	358.176
**13**	-4.124	1.884	1.959	7.805	3.838	231.267	368.075
**14**	-1.116	-0.034	0.431	5.697	5.852	111.498	263.28
**15**	-3.409	1.468	1.579	6.064	3.837	236.891	337.538
**16**	-3.778	1.521	1.538	5.968	3.633	262.659	342.912

Table S4. Predicted medicinal chemistry properties of the compounds

Compounds	QED	SAScore	GASA	Fsp3	MCE-18	NPscore	Lipinski Rule	Pfizer Rule	GSK Rule	Golden Triangle	PAINS	Alarm NMR Rule	BMS Rule	Chelating Rule
**1**	0.229	Easy	Easy	0.286	91.0	2.16	Rejected	Accepted	Rejected	Accepted	1	3	0	1
**2**	0.229	Easy	Easy	0.286	91.0	2.16	Rejected	Accepted	Rejected	Accepted	1	3	0	1
**3**	0.131	Easy	Hard	0.423	122.027	2.421	Rejected	Accepted	Rejected	Rejected	1	3	0	1
**4**	0.131	Easy	Hard	0.423	122.027	2.421	Rejected	Accepted	Rejected	Rejected	1	3	0	1
**5**	0.166	Easy	Hard	0.444	117.692	2.232	Rejected	Accepted	Rejected	Rejected	0	2	0	0
**6**	0.167	Easy	Hard	0.423	118.541	2.349	Rejected	Accepted	Rejected	Rejected	0	2	0	0
**7**	0.265	Easy	Easy	0.462	87.579	2.061	Rejected	Accepted	Rejected	Rejected	0	1	0	0
**8**	0.429	Easy	Easy	0.4	66.048	2.119	Accepted	Accepted	Accepted	Accepted	0	3	0	1
**9**	0.277	Easy	Hard	0.409	93.194	2.165	Rejected	Accepted	Rejected	Accepted	1	3	0	1
**10**	0.247	Easy	Easy	0.286	91.0	2.027	Rejected	Accepted	Rejected	Accepted	1	3	0	1
**11**	0.33	Easy	Hard	0.318	86.966	1.855	Accepted	Accepted	Rejected	Accepted	0	2	0	0
**12**	0.129	Easy	Easy	0.269	80.727	1.294	Rejected	Accepted	Rejected	Rejected	1	3	0	1
**13**	0.129	Easy	Easy	0.269	80.727	1.58	Rejected	Accepted	Rejected	Rejected	1	3	0	1
**14**	0.36	Easy	Hard	0.789	48.059	2.607	Accepted	Accepted	Accepted	Accepted	0	0	0	0
**15**	0.156	Easy	Easy	0.24	81.29	1.349	Rejected	Accepted	Rejected	Rejected	1	3	0	1
**16**	0.156	Easy	Easy	0.24	81.29	1.642	Rejected	Accepted	Rejected	Rejected	1	3	0	1

BMS: Bristol-Myers Squibb; Fsp3: the number of sp^3 hybridized carbons/total carbon count; GASA: graph attention-based assessment of synthetic accessibility; MCE-18: medicinal chemistry 2018; NMR: nuclear magnetic resonance; NPscore: natural product-likeness score; PAINS: pan assay ınterference compounds; QED: quantitative estimate of druglikeness; SAScore: synthetic accessibility score

Table S5. Predicted absorbtion and distribution properties of the compounds

Compounds	Absorbtion									Distribution				
	Caco-2 Permeability (log cm/s)	MDCK Permeability (cm/s)	PAMPA (logPeff)	Pgp inhibitor	Pgp substrate	HIA	F20%	F30%	F50%	PPB	Fu	MRP1 inhibitor	OATP1B1 inhibitor	OATP1B3 inhibitor
**1**	-6.243	-4.996	0.97	0.0	0.144	0.723	0.966	1.0	1.0	84.9%	13.5%	+	+++	+++
**2**	-6.255	-4.959	0.958	0.0	0.094	0.194	0.409	0.999	1.0	84.4%	13.7%	---	+++	+++
**3**	-6.516	-5.067	0.997	0.0	0.277	0.952	0.995	1.0	1.0	81.8%	16.2%	++	++	+++
**4**	-6.53	-5.014	0.993	0.0	0.296	0.874	0.936	1.0	1.0	81.5%	16.8%	--	+++	+++
**5**	-6.307	-5.283	1.0	0.0	0.332	0.956	0.87	0.999	0.999	85.6%	14.2%	++	+	+++
**6**	-6.514	-5.133	1.0	0.0	0.559	0.847	0.935	0.999	0.999	85.3%	12.7%	++	-	+++
**7**	-5.465	-5.058	0.606	0.003	0.075	0.527	0.227	0.997	0.993	75.9%	21.5%	---	+++	+++
**8**	-6.317	-5.168	0.996	0.0	0.001	0.1	0.73	0.978	0.715	59.8%	41.7%	+++	+++	+++
**9**	-6.315	-5.235	0.995	0.0	0.042	0.743	0.472	0.953	0.977	65.6%	33.8%	+	+++	+++
**10**	-6.239	-5.066	0.999	0.0	0.281	0.635	0.922	0.999	0.999	84.6%	12.7%	++	--	+++
**11**	-6.037	-5.211	0.999	0.0	0.201	0.727	0.437	0.971	0.989	86.4%	13.5%	+	+	+++
**12**	-6.31	-5.175	0.83	0.012	0.012	0.177	1.0	1.0	1.0	74.3%	31.1%	--	+++	+++
**13**	-6.397	-5.162	0.96	0.001	0.069	0.189	1.0	1.0	1.0	74.4%	22.7%	--	+++	+++
**14**	-5.719	-5.147	0.948	0.004	0.838	0.814	0.488	0.927	0.984	69.1%	28.9%	-	+++	+++
**15**	-6.666	-5.064	0.997	0.0	0.005	0.296	1.0	1.0	1.0	67.2%	33.4%	---	+++	+++
**16**	-6.708	-5.077	1.0	0.0	0.017	0.213	1.0	1.0	1.0	62.0%	29.3%	---	+++	+++

The prediction probabilities are mapped to six symbols: 0-0.1 (---), 0.1-0.3 (--), 0.3-0.5 (-), 0.5-0.7 (+), 0.7-0.9 (++), and 0.9-1.0 (+++). Fu: fraction unbound in plasma; HIA: human intestinal absorption; MDCK: Madin–Darby Canine Kidney cells; MRP1: multidrug resistance protein 1; OATP: organic anion transporting polypeptide; PPB: plasma protein binding; PAMPA: parallel artificial membrane permeability assay; Pgp: P-glycoprotein

Table S6. Predicted metabolism and excretion properties of the compounds

Compounds	Metabolism													Excretion	
	CYP1A2 inhibitor	CYP1A2 substrate	CYP2C19 inhibitor	CYP2C19 substrate	CYP2C9 inhibitor	CYP2C9 substrate	CYP2D6 inhibitor	CYP2D6 substrate	CYP3A4 inhibitor	CYP3A4 substrate	CYP2B6 substrate	CYP2C8 inhibitor	HLM Stability	CL plasma (ml/min/kg)	$T_{1/2}$ (h)
**1**	---	---	---	---	---	---	---	---	--	---	---	+++	++	3.496	2.842
**2**	---	---	---	---	---	---	---	---	++	---	---	+++	++	3.528	3.634
**3**	---	---	---	---	---	---	---	---	+++	---	---	+++	++	2.448	3.003
**4**	---	---	---	---	---	---	---	---	++	---	---	+++	++	2.357	3.401
**5**	---	---	---	---	---	---	---	---	--	---	---	--	---	2.69	3.066
**6**	---	---	---	---	---	---	---	---	++	---	---	++	--	2.34	3.196
**7**	---	--	---	--	---	---	---	---	---	---	---	---	---	2.426	3.634
**8**	---	---	---	---	---	---	---	---	---	---	---	---	---	2.87	3.178
**9**	---	---	---	---	---	---	---	---	---	---	---	++	---	4.503	3.649
**10**	--	---	---	---	---	---	---	---	-	---	---	+++	---	4.426	4.489
**11**	---	---	---	---	---	---	---	---	--	---	---	++	---	4.436	4.071
**12**	---	---	---	---	---	---	---	---	---	---	---	+++	--	8.529	2.125
**13**	---	---	---	---	---	---	---	---	-	---	---	+++	-	10.471	2.363
**14**	---	---	---	---	---	---	---	---	---	-	---	-	-	2.311	2.092
**15**	---	---	---	---	---	---	---	---	---	---	---	+++	---	5.225	2.505
**16**	---	---	---	---	---	---	---	---	---	---	---	+++	---	4.139	2.748

The prediction probabilities are mapped to six symbols: 0-0.1 (---), 0.1-0.3 (--), 0.3-0.5 (-), 0.5-0.7 (+), 0.7-0.9 (++), and 0.9-1.0 (+++). CL: clearance; CYP: cytochrome; HLM: human liver microsomal; $T_{1/2}$: half-life.

Hyperoside **(1)** ESI-MS m/z 465 $[M+H]^+$; $[C_{21}H_{20}O_{12}]$;^{1}H-NMR (MeOD, 400 MHz) δ = 7.74 (1H, d, *J* = 2.0 Hz, H-6'), 7.48 (1H, dd, *J* = 8.4, 2.4 Hz, H-5'), 6.76 (1H, d, *J* = 8.8 Hz, H-2'), 6.30 (1H, d, *J* = 2 Hz, H-8), 6.10 (1H, d, *J* = 2 Hz, H-6), 5.07 (1H, d, *J* = 7.6 Hz, H-1″), 3.75-3.36 (6H, m, H-2″-6″);^{13}C-NMR (MeOD, 125 MHz) 179.64 (C-4), 166.18 (C-7), 163.11 (C-5), 158.89 (C-9), 158.54 (C-2'), 150.06 (C-4'), 145.92 (C-3'), 135.86 (C-3), 123.02 (C-6'), 122.95 (C-1'), 117.87 (C-5'), 116.18 (C-2), 105.72 (C-10), 105.47 (C-1″), 99.99 (C-6), 94.81 (C-8), 77.28 (C-5″), 75.17 (C-3″), 73.26 (C-2″), 70.11 (C-4″), 62.02 (C-6″).

Isoquercetin **(2)** ESI-MS m/z 465 $[M+H]^+$; $[C_{21}H_{20}O_{12}]$;^{1}H-NMR (MeOD, 400 MHz) δ = 7.73 (1H, d, *J* = 2.0 Hz, H-6'), 7.60 (1H, dd, *J* = 8.5, 2.0 Hz, H-5'), 6.88 (1H, d, *J* = 8.5 Hz, H-2'), 6.40 (1H, d, *J* = 2.0 Hz, H-8), 6.21 (1H, d, *J* = 2.0 Hz, H-6), 5.26 (1H, d, *J* = 9.0 Hz, H-1″), 3.750-3.322 (6H, m, H-2″-6″);^{13}C-NMR (MeOD, 125 MHz) 178.07 (C-4), 164.66 (C-7), 161.62 (C-5), 157.60 (C-9), 157.05 (C-2'), 148.44 (C-4'), 144.49 (C-3'), 134.22 (C-3), 121.79 (C-6'), 121.67 (C-1'), 116.16 (C-5'), 114.60 (C-2), 104.27 (C-10), 102.93 (C-1″), 98.50 (C-6), 93.32 (C-8), 76.97 (C-5″), 76.71 (C-3″), 74.33 (C-2″), 69.81 (C-4″), 61.15 (C-6″).

Quercetin 3-*O*-*β*-apiofuranosyl-(1→2)-*β*-galactopyranoside **(3)** ESI-MS m/z 619.86 $[M+Na]^+$ δ 7.53 (1H, dd, *J*= 8.8, 2.0 H-6'), 7.60 (1H, d, *J*= 2.0, H-2'), 6.77 (1H, *J*= 8.4, H-5'), 6.27 (1H, d, *J*= 1.6, H-8), 6.07 (1H, d, *J*= 2.0, H-6), 5.39 (1H, d, *J*= 8.0, H-1″), 5.35 (1H, *J*= 1.6, H-1‴), 3.94 (1H, m, H-2‴), 3.92 (2H, s, H-4‴), 3.84 (1H, m, H-2″), 3.71 (1H, m, H-4″), 3.63-3.51 (2H, m, H-5‴), 3.54 (1H, m, H-3″), 3.48-3.45 (2H, m, H-6″), 3.32 (1H, H-5″). ^{13}C NMR: δ 179.93 (C-4), 165.66 (C-7), 162.91 (C-5), 158.25 (C-9), 49.84 (C-4'), 145.53 (C-3'),123.35 (C-1'), 123.19 (C-6'), 117.60 (C-2'), 116.15 (C-5'), 110.75 (C-1‴), 101.33 (C-1″), 99.78 (C-6), 94.38 (C-8), 80.95 (C-3‴), 78.05 (C-2‴), 77.07 (C-5″), 76.77 (C-2″), 75.35 (C-4‴), 75.26 (C-4″), 70.65 (C-4″), 66.21 (C-5‴), 62.01 (C-6″).

Quercetin 3-*O*-*β*-apiofuranosyl-(1→2)-*β*-glucopyranoside **(4)** ESI-MS m/z 619.93 $[M+Na]^+$ δ 7.53 (1H, dd, *J*= 8.8, 2.0 H-6'), 7.53 (1H, brs, H-2'), 6.76 (1H, *J*= 8.0, H-5'), 6.27 (1H, d, *J*= 2.0, H-8), 6.08 (1H, *J*= 2.0, H-6), 5.44 (1H, d, *J*= 7.6, H-1″), 5.37 (1H, *J*= 1.6, H-1‴), 3.93 (1H, m, H-2‴), 3.93-3.58 (2H, m, H-4‴), 3.59 (1H, m, H-4‴), 3.57-3.30 (2H, m, H-6″), 3.56 (1H, m, H-2″), 3.63-3.51 (2H, m, H-5‴), 3.42 (1H, m, H-5″), 3.21 (1H, m, H-4″), 3.07 (1H, m, H-3″). ^{13}C NMR: δ 178.09 (C-4), 164.38 (C-7), 161.83 (C-5), 156.96 (C-9), 156.96 (C-2), 148.23 (C-4'), 144.46 (C-3'), 133.34 (C-3), 122.05 (C-1'), 121.98 (C-6'), 115.74 (C-2'), 114.64 (C-5'), 109.16 (C-1‴), 104.03 (C-10), 99.38 (C-1″), 98.32 (C-6), 93.20 (C-8), 79.51 (C-3‴), 77.55 (C-2″), 77.22 (C-3″), 76.90 (C-5″), 76.67 (C-2‴), 74.17 (C-4‴), 70.09 (C-4″), 64.93 (C-5‴), 61.19 (C-6‴).

7-Methoxyapigenin 6-C-[*β*-apiofuranosyl-(1→2)]-*β*-glucopyranoside **(5):** ESI-MS m/z 578.24 $[M - H]^-$ ^{1}H NMR (D_3OD; 400 MHz): δ 7.86 (2H, d, *J*= 9.2, H-2' and H-6'), 6.92 (2H, d, *J*= 8.4, H-3' and H-5'), δ 6.71 (1H, s, H-8), δ 6.63 (1H, s, H-3), 5.28 (1H, brs, H-1‴), 4.85/4.87 (1H, d, *J*= 10.0, H-1″), 4.52/4.37 (1H, t, *J*= 9.2, H-2″), 3.87 (1H, m, H-6″), 3.70 (1H, m, H-6″), 3.76 (1H, d, *J*= 2.4 H-2‴), 3.58 (1H, m, H-3″), 3.43/3.39 (1H, t, *J*=9.2, H-4″), 3.35 (1H, m, H-5″), 3.25 (1H, m, H-5‴), 3.15 (1H, m, H-5‴), 3.22 (1H, m, H-4‴), 2.71/2.56 (1H, d, *J*=9.6 H-4‴), 3.93/3.92 (3H, s, 7- OCH_3). ^{13}C NMR: δ 184.18/183.89 (C-4), 167.24/165.32/ (C-7), 166.39 /166.37 (C-2), 162.93/162.89 (C-4'), 162.02/161.01 (C-5), 159.23/159.03 (C-9), 129.60/129.58 (C-2' and C-6'), 123.01/122.95 (C-1'), 117.09 (C-3'and C-5'), 111.01/110.92 (C-1‴), 110.69/110.59 (C-6), 106.14/105.70 (C-10), 104.25/104.17 (C-3), 92.02/91.21 (C-8), 82.51 (C-5″), 81.05 (C-3″), 80.59 (C-3‴), 77.81/77.73 (C-2‴), 76.72/76.45 (C-2″), 74.92/74.80 (C-4‴), 73.33/72.88 (C-1″), 72.28/72.00 (C-4″), 66.01/65.84 (C-5‴), 63.44/63.33 (C-6″), 57.01/56.81 (OCH_3) (Ma et al., 1998; Feng et al., 2011).

Apigenin 6-C-[*β*-apiofuranosyl-(1 → 2)]-*β*-glucopyranoside **(6)** ESI-MS m/z 588.18 $[M+Na]^+$, 564.20 $[M - H]^-$ ^{1}H NMR (D_3OD; 400 MHz): δ 7.68 (2H, d, *J*= 8.4, H-2' and H-6'), 6.77 (2H, d, *J*= 8.4, H-3' and H-5'), δ 6.35 (1H, s, H-8), δ 6.19 (1H, s, H-3), δ 5.31 (1H, brs, H-1‴), 4.88 (1H, d, *J*= 7.5, H-1″), 3.72 (1H, m, H-6″), 3.65

(1H, m, H-6″), 5.28 (1H, brs,), 3.2 (2H, m, H-4‴), 3.10-3.15 (1H, m, H-5‴). ^{13}C NMR: δ 127.48 (C-2'-C-6'), 116.13 (C-3'-5'), 109.18 (C-1‴), 101.30 (C-3), 95.55 (C-8), 73.65 (C-4‴), 64.92 (C-5‴), 61.33 (C-6″).

Dihydrodehydrodiconiferyl alcohol 4-*O*-*β*-glucopyranoside **(7)** ESI-MS m/z 546.13 [M+Na]$^+$ ^{1}H NMR (D_3OD; 400 MHz): δ 7.13 (1H, d, *J*= 8.0, H-5), 7.02 (1H, d, *J*= 1.6, H-2), 6.92 (1H, dd, *J*= 2.0, 8.0, H-6), 6.72 (1H, brs, H-2'), 6.71 (1H, brs, H-6'), 5.55 (1H, d, *J*= 6.0, H-7), 4.8 (1H, under solvent peak, H-1″), 3.85 (3H, s, OCH_3 at C-3'), 3.82 (3H, s, OCH_3 at C-3), 3.84 (1H, m, H-6″), 3.68 (1H, m, H-6″), 3.56 (2H, t, *J*=6.8, C-9'), 3.50-3.30 (4H, m, H-2″-H-5″). 3.46 (1H, m, H-8), 2.62 (2H, dd, *J*= 4.0, 8.0, C-7'), 1.80 (2H, m, H-8'). ^{13}C NMR: δ 150.95 (C-3), 147.63 (C-4), 147.51 (C-4'), 145.21 (C-3'), 138.37 (C-1), 137.11 (C-1'), 129.59 (C-5'), 119.38 (C-6), 118.03 (C-5), 117.95 (C-6'), 114.15 (C-2'), 111.13 (C-2), 102.77 (C-1″), 88.48 (C-7), 78.22 (C-3″), 77.86 (C-5″), 74.92 (C-2″), 71.34 (C-4″), 65.08 (C-9), 62.50 (C-6″), 62.24 (C-9'), 56.77 and 56.70 (2xOCH_3), 55.71 (C-8), 35.86 (C-8'), 32.93 (C-7').

Cichoriin **(8)** ESI-MS m/z 339.91 [M - H]$^-$, m/z 341.35 [M+H]$^+$ ^{1}H NMR (CD_3OD; 400 MHz): δ 7.82 (1H, d, *J* = 10.0 Hz, H-4), 7.20 (1H, s, H-8), 7.03 (1H, s, H-5), 6.28 (1H, d, *J* = 9.6 Hz, H-3), 4.97 (1H, d, *J* = 7.2 Hz, H-1'), 3.29-3.94 (m, 6H, H-2`-6`).

7-*O*-Methly isoorientin **(9)** ESI-MS m/z 486.01 [M+Na]$^+$, m/z 461.74 [M-H]$^-$ ^{1}H NMR (CD_3OD; 400 MHz): δ 7.47 (1H, *J*= 8.0, H-6'), 7.46 (1H, brs, H-2'), 6.91 (1H, d, *J*=8.1 Hz H-5'), 6.79/6.78 (1H, s, H-8), δ 6.76/6.74 (1H, s, H-3), 4.58/4.56 (1H, d, *J*= 9.6, H-1″), 4.18/3.99 (1H, t, *J*= 9.0, H-2″), 3.71-3.05 (5H, m, H-3″-6″), 3.90/3.879 (3H, s, 7- OCH3). ^{13}C NMR: δ 182.63/182.31 (C-4), 164.55/164.39 (C-7), 164.21 (C-2), 160.80/160.05 (C-5), 157.28/157.17 (C-9), 150.36 (C-4'), 146.29 (C-3'), 121.77 (C-1'), 119.53 (C-6'), 116.45 (C-5'), 113.97 (C-2'), 110.12 (C-6), 105.07/104.56 (C-10), 103.54 (C-3), 91.38/90.54 (C-8), 82.35/82.17 (C-5″), 79.55 (C-3″), 73.29/73.04 (C-1″), 71.38/71.30 (C-4″), 70.74/70.08 (C-2″), 62.22 (C-6″), 56.96/56.72 (OCH_3).

Isoorientin **(10)** ESI-MS m/z 447.85 [M-H]$^-$ ^{1}H NMR (D3OD; 400 MHz): δ 7.36 (1H, *J*= 7.2, H-6'), 7.35 (1H, brs, H-2'), 6.88 (1H, *J*= 8.4, H-5'), 6.53 (1H, s, H-8), δ 6.47 (1H, s, H-3), 4.9 (1H, d, *J*= 9.6, H-1″), 4.16 (1H, t, *J*= 9.2, H-2″), 3.89 (1H, dd, *J*=2.0, 11.2, H-6″), 3.73 (1H, dd, *J*=5.2, 12.0, H-6″), 3.49-3.39 (3H, m, H-3″, H-4″, H-5″). ^{13}C NMR: δ 184.02 (C-4), 166.27 (C-2), 164.94 (C-7), 162.06 (C-5), 158.72 (C-9), 151.09 (C-4'), 147.07 (C-3'), 123.55 (C-1'), 120.34 (C-6'), 116.81 (C-5'), 114.17 (C-2'), 109.20 (C-6), 105.21 (C-10), 103.92 (C-3), 95.21 (C-8), 82.66 (C-5″), 80.15 (C-3″), 75.32 (C-1″), 72.60 (C-2″), 71.81 (C-4″), 62.90 (C-6″).

Swertisin **(11)** ESI-MS m/z 447.92 [M+H]$^+$ m/z 445.85 [M-H]$^-$ δ 7.98 (2H, d, *J*= 8.4, H-2' and H-6'), 6.94 (2H, d, *J*= 8.8, H-3' and H-5'), δ 6.85/6.84 (1H, s, H-8), δ 6.83 (1H, s, H-3), 4.60/4.58 (1H, d, *J*= 9.2, H-1″), 4.00/3.71 (1H, t, *J*= 9.2, H-2″), 3.80-3.00 (1H, m, H-3″, H-4″, H-5″, H-6″), 3.90/3.87 (3H, s, 7- OCH_3). ^{13}C NMR: δ 182.74/182.41 (C-4), 165.43/164.37 (C-7), 164.22 (C-4'), 161.76 (C-2), 160.76/160.03 (C-5), 157.31/157.19 (C-9), 129.02 (C-2'-C-6'), 121.45 (C-1'), 116.46 (C-3'-5'), 110.16/110.09 (C-6), 105.07/104.56 (C-10), 103.51 (C-3), 91.50/90.66 (C-8), 82.33/82.14 (C-5″), 79.54/79.49 (C-3″), 73.29/73.05 (C-2″), 71.37/71.28 (C-1″), 70.73/70.09 (C-4″), 62.21 (C-6″), 56.96/56.72 (OCH3).

3,5-*O*-Dicaffeoylquinic acid methyl ester **(12)** ESI-MS m/z 529.85 $[M-H]^-$ ^{1}H-NMR (400 MHz, MeOH-d4) δ: 7.62 (1H, d, *J*=16.4 Hz, H-7'), 7.58 (1H, d, *J*=16.4 Hz, H-7''), 7.13/7.14 (2H, d, *J*=2.0 Hz, H-2', 2''), 7.06,7.05 (2H, dd, *J*=2.0, 8.0 Hz H-6', 6''), 6.86, 6.86 (2H, d, *J*=7.6 Hz, H-5', 5''), 6.36 (1H, d, *J*=16 Hz, H-8'), 6.27 (1H, d, *J*=16 Hz, H-8''), 5.34 (1H, m, H-5), 5.31 (1H, m, H-3), 3.90 (1H, dd, *J*=3.2, 9.6 Hz H-4), 3.69 (3H, s, OMe), 2.26-1.95 (4H, m, H-2, 6

4,5-*O*-Dicaffeoylquinic acid methyl ester **(13)** ESI-MS m/z 529. $[M-H]^-$ ^{1}H-NMR (500 MHz, MeOH-d4) δ: 7.58 (1H, d, *J*=16 Hz, H-7'), 7.53 (1H, d, *J*=16 Hz, H-7''), 7.09 (2H, d, *J*=2.0 Hz, H-2' or 2''), 7.07 (2H, d, *J*=2.4 Hz, H-2' or 2''), 6.98 (2H, dd, *J*=2.0, 8.4 Hz, H-6' or 6''), 7.00 (2H, dd, *J*=2.0, 8.4 Hz, H-6' or 6''), 6.82 (2H, d, *J*=8.4 Hz, H-5' and 5''), 6.29 (1H, d, *J*=16 Hz, H-8'), 6.21 (1H, d, *J*=16 Hz, H-8''), 5.58 (1H, m, H-5), 5.08 (1H, dd, *J*=3.2; 9.6 Hz -4), 4.33 (1H, m, H-3), 3.72 (3H, s, OMe), 2.20-1.91 (4H, m, H-2, 6).

Staphylionoside E **(14)** ESI-MS (negative and positive) *m/z* 387.25 $[M–H]^-$ and 411.25 $[M+Na]^+$. (CH_3OH); ^{1}H-NMR (400 MHz, CD_3OD) 5.94 (1H, d, *J* = 16.0 Hz, H-7), 5.41 (1H, dd, *J* = 16.0, 6.0 Hz, H-8), 4.40 (1H, d, *J* = 7.8 Hz, H-1′), 4.20 (1H, m, H-9), 3.98 (1H, d, *J* = 3.3 Hz, H-4), 3.88 (1H, ddd, *J* = 12.8, 3.6, 3.6 Hz, H-3), 3.75 (1H, dd, *J* = 12.0, 1.6 Hz, H-6′a), 3.57 (1H, dd, *J* = 12.0, 5.2 Hz, H-6′b), 3.28 (1H, m, H-5′) 3.20 (1H, dd, *J* = 8.0, 8.0 Hz, H-4′), 3.12 (1H, dd, *J* = 7.6, 8.0 Hz, H-2′), 1.78 (1H, m, H-2a), 1.47 (1H, ddd, *J* = 12.0, 3.2, 1.6 Hz, H-2b), 1.74 (3H, s, H-13), 1.16 (3H, d, *J* = 6.0 Hz, H-10), 0.99 (3H, s, H-12), 0.93 (3H, d, H-11),^{13}C-NMR 141.66 (C-6), 139.29 (C-8), 126.55 (C-5), 125.32 (C-7), 101.29 (C-1′), 76.72 (C-3′), 76.66 (C-5′), 74.69 (C-3), 73.96 (C-2′), 70.20 (C-4′), 68.75 (C-4), 68.07 (C-9), 61.32 (C-6′), 38.58 (C-2), δ 36.46 (C-1), 28.87 (C-11), 26.33 (C-12), 22.45 (C-10), 18.50 (C-13).

3,5-*O*-Dicaffeoylquinic acid **(15)** was obtained as a white powder. $C_{25}H_{24}O_{12}$; ESI-MS m/z: 515.89 $[M-H]^-$. ^{1}H NMR (400 MHz, CD_3OD) δ: 7.61 (1H, d, *J* = 16.0 Hz, H-7″), 7.57 (1H, d, *J* = 16.0 Hz, H-7ʹ), 7.06 (2H, d, *J* = 2.0 Hz, H-2ʹ/ H-2″), 7.05 (2H, d, *J* = 2.0 Hz, H-2ʹ/H-2″), 6.96 (2H, dd, *J* = 8.4, 2.0 Hz, H-6ʹ, H-6″), 6.77 (2H, d, *J* = 8.0 Hz, H-5ʹ, H-5″), 6.37 (1H, d, *J* = 16.0 Hz, H-8″), 6.28 (1H, d, *J* = 16.0 Hz, H-8ʹ), 5.45 (1H, m, H-5), 5.40 (1H, dd, *J* = 4.0; 8.4 Hz, H-3), 3.943 (1H, dd, *J* = 3.2; 8.4 Hz, H-4), 2.29 (1H, dd, *J* = 14.4; 3.6 Hz, H-6eq), 2.16 (2H, d, *J* = 6.4, H-2), 2.11 (1H, dd, *J* = 14.0, 5.2 Hz, H-6ax); ^{13}C NMR (150.9 MHz, CD3OD) δ: 179.90 (C-7), 169.15 (C-9ʹ or 9″), 168.66 (C-9ʹ or 9″), 149.59 (C-4ʹ), 149.47 (C-4″), 147.12 (C-7ʹ), 146.96 (C-7″), 146.81 (C-3ʹ), 146.79 (C-3″), 128.02 (C-1ʹ), 127.85 (C-1″), 123.04 (C-6ʹ), 123.04 (C-6″), 116.49 (C-5ʹ), 116.49 (C-5″), 115.81 (C-8ʹ), 115.33 (C-8″), 115.20 (C-2ʹ), 115.14 (C-2″), 75.10 (C-1), 73.45 (C-5), 72.27 (C-3), 71.85 (C-4), 39.17 (C-2), 36.79 (C-6).

4,5-*O*-Dicaffeoylquinic acid **(16)**: ESI-MS *m/z* 515.82 $[M–H]^-$ ($C_{25}H_{24}O_{12}$).^{1}H-NMR (in CD_3OD, 600 MHz) *δ* 7.57 (1H, d, *J* = 15.6 Hz, H-7′ or H-7′′), 7.49 (1H, d, *J* = 15.6 Hz, H-7′ or H-7′′), 6.91 (1H, d, *J* = 1.6 Hz, H-2′ or H-2′′), 6.88 (1H, d, *J* = 1.6 Hz, H-2′ or H-2′′), 6.89 (1H, dd, , *J*= 1.6; 8.0 Hz, H-6′ or H-6′′), 6.87 (1H, dd, , *J*= 1.6; 8.0 Hz, H-6′ or H-6′′), 6.73 (1H, d, *J*=8.0 Hz, H-5′ or H-5′′), 6.72 (1H, d, *J*= 8.0 Hz, H-5′ or H-5′′), 6.17 (1H, d, *J* = 15.6 Hz, H-8′ or H-8′′), 6.08 (1H, d, *J* = 15.6 Hz, H-8′ or H-8′′), 5.56 (1H, m, H-5), 5.02 (1H, dd, *J*= 2.8; 9.6 H-4), 4.26 (1H, brs, H-3), 2.17-2.10 (2H, m, H-6 and 2H, m, H-2); ^{13}C-NMR *δ* 168.69 (C-9′ or 9′′), 168.51 (C-9′ or 9′′), 149.74 (C-4′ and 4′′), 147.76 (C-7′ or 7′′), 147.60 (C-7′ or 7′′), 146.85 (C-3′ or 3′′), 146.82 (C-3′ or 3′′), 127.79 (C-1′ or 1′′), 127.73 (C-1′ or 1′′), 123.22 (C-6′ and 6′′), 116.55 (C-5′ and 5′′), 115.23 (C-2′ and 2′′), 114.87 (C-8′ and 8′′), 77.40 (C-1), 76.55 (C-2), 70.20 (C-3), 69.26 (C-5), 40.17 (C-6), 38.57 (C-4).
